# Determinants of Parental Emotion Socialization Behaviors: Insights From a Large‐Scale Population‐Based Family Study

**DOI:** 10.1111/sjop.70082

**Published:** 2026-02-18

**Authors:** Yvonne Severinsen, Eivind Ystrom, Nikolai Haahjem Eftedal, Egil Nygaard, Mona Bekkhus, Karine Maria Porpino Viana, Stella Tsotsi, Evalill Bølstad, Rune Flaaten Bjørk

**Affiliations:** ^1^ Department of Psychology, Promenta Research Center University of Oslo Oslo Norway; ^2^ Department of Child Health and Development Norwegian Institute of Public Health Oslo Norway; ^3^ Centre for Research on Equality in Education, Faculty of Educational Sciences University of Oslo Oslo Norway; ^4^ Department of Social Work, Child Welfare and Social Policy Oslo Metropolitan University Oslo Norway; ^5^ Department of Nursing and Health Promotion Oslo Metropolitan University Oslo Norway; ^6^ Section for Health, Developmental‐ and Personality Psychology, Department of Psychology University of Oslo Oslo Norway; ^7^ Section for Clinical Psychology, Department of Psychology University of Oslo Oslo Norway; ^8^ Vestre Viken Helseforetak Drammen Norway

**Keywords:** emotion socialization, intergenerational transmission, parenting, parenting process models

## Abstract

Parental emotion socialization behaviors (ESBs) are essential for child development and are typically understood as influenced by factors related to three domains: the parent, the child, and the family context. However, it remains unclear how parents' childhood experiences with ESBs should be represented within these conceptual frameworks. Addressing the complexity of ESBs, the present study examined predictors across three theoretically derived domains and additionally included parents' recollected ESBs as a parent‐level factor. Questionnaire data were collected from a large population‐based cohort of Norwegian mothers and fathers (*n* = 4207; 2460 mothers) with children aged 3 to 13 years (*M* = 7.4 years). Supportive and non‐supportive ESBs were examined separately through hierarchical regression models incorporating parent, context, and child factors. The three domains collectively accounted for 28% of the variance in supportive ESBs and 29% in non‐supportive ESBs. The results clearly emphasized parental factors, with parent gender and recollected ESBs being the most influential predictors. Other parent factors, such as perceived parenting stress, alcohol use, and the personality traits agreeableness and openness, also showed unique effects across both types of ESBs. Among contextual factors, education, income, and country of birth predicted non‐supportive ESBs but not supportive ESBs. Child age and birth order were linked with supportive ESBs, whereas only child age predicted non‐supportive ESBs. Findings highlight the importance of parent characteristics, particularly gender and recollected emotion socialization from the family of origin, in predicting current parental ESBs beyond contextual and child‐related factors.

## Summary

- Predictors from the parent, child, and family context domains jointly accounted for 28%–29% of the variance in supportive and non‐supportive parental emotion socialization behavior.
- Parents' characteristics, particularly gender and recollected experiences of emotion socialization in their family of origin, were the strongest predictors.
- Contextual and child‐related factors made smaller contributions, mainly to non‐supportive behaviors.
- Findings underscore the role of parents' developmental history and individual characteristics in understanding current emotion socialization practices.

Research on parental emotion socialization behaviors (ESBs) highlights their significant role in influencing children's development (Eisenberg 2020; Hajal and Paley 2020; Morris et al. 2022), with parental responses to children's emotions predicting a variety of child outcomes, including psychopathology (Godleski et al. 2020; Johnson et al. 2017), externalizing behavior (Bjørk et al. 2024; Frogley et al. 2023; McKee et al. 2022), emotion regulation (De Raeymaecker and Dhar 2022; Lunkenheimer et al. 2007; Sosa‐Hernandez et al. 2020), as well as socioemotional competence (Thompson et al. 2020). Thus, it is crucial to understand the sources of variation in parental ESBs.

## 1 Parental ESBs

Parental ESBs are a context‐specific parenting practice defined as behaviors that shape children's experience, regulation, expression, and comprehension of emotions (Zahn‐Waxler 2010), with the goal of helping individuals successfully adapt and function in their social environment (Hofmann and Doan 2018). These behaviors are shaped by cultural norms and parents' beliefs about the role of emotions, guided by both implicit and unconscious rules, as well as explicit conscious strategies (Gottman et al. 1996).

Eisenberg et al. (1998) provided a framework for emotion socialization, which, along with Gottman et al.'s (1996) parental meta‐emotion philosophy, has guided research in this field. Emotion socialization theory suggests that children learn about emotions primarily through their interactions with parents, including parental reactions to emotions, family discussions about emotions, and the ways parents express their own emotions (Eisenberg et al. 1998). ESBs are typically operationalized as supportive (i.e., validating, encouraging expression, and problem‐solving around emotions) or non‐supportive (i.e., punitive or dismissive towards emotions; De Raeymaecker and Dhar 2022; Fabes et al. 2001). Supportive ESBs help children understand and express emotions in a socially appropriate manner. In contrast, when parents respond with dismissal or punishment, as examples of unsupportive ESBs, children are deprived of this crucial learning opportunity (Gottman et al. 1996). Research on emotion socialization has focused on parents' reactions to children's negative emotions, recognizing that coping with negative emotions represents a significant challenge for both parents and children (Kiff et al. 2011; Nelson et al. 2009).

## 2 Predictors of Parental ESBs

Recognizing that ESBs are part of a broader concept of supportive and non‐supportive parenting styles (Eisenberg et al. 1998), as well as the limitations in research focusing specifically on parental ESBs, this study relies on literature related to the influential process model of parenting in general (Belsky 1984; Taraban and Shaw 2018) and the well‐established model of parental ESBs (Eisenberg 2020; Eisenberg et al. 1998). Parenting process models typically identify three main sources of influence on parenting behavior: the family context, the parent, and the child (Belsky 1984; Eisenberg et al. 1998). Empirical evidence on variation in parental ESBs related to key predictors within each of these three domains will be summarized.

### 2.1 Family Context Factors

Taraban and Shaw's (2018) detailed revision of empirical data for Belsky's parenting model indicated that the strongest support was found in the family context, highlighting the role of socioeconomic status (SES) and marital quality, above parents' characteristics. Evidence suggests that higher SES correlates with increased use of supportive parenting strategies, and low SES with more use of non‐supportive parenting (Lugo‐Candelas et al. 2015; Shaffer et al. 2012). However, findings on ESBs are heterogenous and contradictory; some studies found no evidence of association between education and ESBs (Dixon‐Gordon et al. 2020) or between income and ESBs (Baker et al. 2011), and, contrary, McKee et al. (2022) found parents with higher levels of education to be less emotion‐supportive, and parents with lower income to be more supportive than parents with high‐income (McKee et al. 2022). Additionally, a recent study profiling parents of children aged 3 to 13 (*n* = 869) found no evidence linking SES to the parent's ESBs profile (King et al. 2023). Notably, cited research on SES has used risk or convenience samples, and given both a lack of population‐based samples and conflicting findings, the impact of SES on ESBs remains unclear.

Belsky considered the marital relationship to be the most essential support system for parents (Belsky 1984), and meta‐analyses have documented a positive association between relationship quality and supportive parenting in general (Conger et al. 2013; Erel and Burman 1995; Taraban and Shaw 2018). In line with these findings, research on parental ESBs suggests a positive association between the quality of marital relations and supportive ESBs (Wong et al. 2009), but research is limited, although Nelson et al. (2009) found marital dissatisfaction to predict less supportive ESBs for fathers, but not for mothers. Regarding family structure, Baker et al. (2011) found a higher number of siblings in the family to be associated with increased use of non‐supportive ESBs in mothers. Cross‐cultural research on parental ESBs suggests that ESBs vary across cultures (for a review, see Raval and Walker 2019). However, there is still a lack of population‐based studies sampling participants from diverse socioeconomic and cultural backgrounds (Thompson et al. 2020).

### 2.2 Parent Factors

The individual characteristics of parents impact their parenting (Bornstein 2016), and among the three domains included in the parenting process model (i.e., context, parent, and child), Belsky (1984) considered the parent as the most crucial element. In addition to specific demographic factors (i.e., gender and age), the parents' psychological characteristics encompass unique internal processing styles and subjective perceptions that can influence parenting (Plomin 2023). Psychological factors such as anxiety, depression, alcohol use, stress, and personality traits are conceptually and empirically interrelated, often co‐occurring and mutually reinforcing. Given this complex interdependence, exploring individual characteristics both collectively and individually allows for rich insights into both unique and shared contributions.

Initial research on parental ESBs and gender found mothers to be more emotion‐supportive in interactions with their children compared to fathers (for reviews, see Root and Denham 2010; Zeman et al. 2010), a pattern supported in more recent studies (Baker et al. 2011; McKee et al. 2022; Yaffe 2023). Demographic factors such as age may also influence ESBs. Rossi's (1980) “timing‐of‐events” model assumes an optimal age for having children, with both early and late parenthood posing challenges. Although research on parent age and ESBs is limited, studies have found that younger maternal age is associated with increased use of non‐supportive ESBs (Scott and Hakim‐Larson 2021; Wong et al. 2009).

Regarding the psychological characteristics of parents, Belsky's original model (Belsky 1984) identifies parents' developmental history as a key predictor of parenting, a notion emphasized in clinical parenting interventions, which place great importance on recollected childhood experiences as formative of current parenting practices (Buston et al. 2022). Initially, research on parents' developmental history focused on parents' history of maltreatment and harsh parenting, and a growing body of research supports the idea that parenting practices are transmitted from one generation to the next (for a review on intergenerational transmission of parenting, see Belsky et al. 2009; Conger et al. 2009; Kerr and Capaldi 2019; Rothenberg 2019). The association between parenting across generations persists when examining recollections of parenting in one's family of origin (Huffhines et al. 2024; Leerkes et al. 2020), and is documented by both self‐reports (Capaldi et al. 2008) and observations (Conger et al. 2013). For instance, Leerkes and Siepak (2006) observed the reactions of 440 students to child distress and found that a history of parental emotional rejection correlated negatively with accurate identification of emotions and positively with negative attributions, amusement, and neutral responses to child distress. Similarly, a longitudinal study revealed that mothers' recollections of non‐supportive ESBs were negatively associated with self‐efficacy in managing toddlers' distress (Cao et al. 2023).

The association between parental mental health and parenting practices is well‐established in the literature (Zahn‐Waxler 2010), with anxiety and depression receiving the most research attention: a focus justified by being the most prevalent symptoms of mental health issues (World Health Organization 2025). The majority of research on parental psychopathology and parenting practices has centered on maternal depression (Breaux et al. 2022), finding a consistent relationship between maternal depression and negative and coercive parenting practices (Lovejoy et al. 2000). Similarly, research on ESBs has consistently demonstrated that elevated levels of psychopathology are associated with an increase in non‐supportive responses to children's negative emotions (Breaux et al. 2022; Godleski et al. 2020), although it should be noted that these findings were observed within risk samples.

Alcohol problems have been shown to have a myriad of effects on parenting behaviors, ranging from inconsistency and lack of discipline (Keller et al. 2005) to more severe forms of neglectful or abusive parenting (Freisthler and Gruenewald 2013). Research specifically on alcohol use and ESBs has also demonstrated a link between higher alcohol consumption and greater use of non‐supportive ESBs (Amundsen et al. 2023; Godleski et al. 2020).

Parenting stress has received increasing attention due to its relationship with parenting practices and child development (Louie et al. 2017). Parental stress refers to the unique pressure that arises directly from parenting responsibilities, setting it apart from other stressors that may impact parenting practices (Berry and Jones 1995), and research suggests that parental stress negatively relates to parents' socialization practices (Nelson et al. 2023).

Personality is also acknowledged as a crucial factor in shaping parenting behavior (Belsky 1984; Prinzie et al. 2009; Taraban and Shaw 2018), and can influence parenting in multifaceted ways: directly, such as through tendencies towards aggression or warmth, and indirectly, such as through the selection of a partner, the influence on the family's emotional climate, and the social network.

Available empirical evidence on ESBs and personality suggests an association, with the most distinct patterns emerging for openness and agreeableness, both positively associated with supportive ESBs (Hughes and Gullone 2010), while results regarding neuroticism and ESBs are inconsistent (Cabecinha‐Alati et al. 2020).

### 2.3 Child Factors

Children enter the world with a unique constitution, which in turn can evoke parental behaviors, giving rise to “child effects” on parenting (e.g., Kiff et al. 2011; Morris et al. 2007). Two genetically informed meta‐analyses concluded that between 23% and 40% of the variation in parenting can be explained by child genetic effects (Avinun and Knafo 2014; Klahr and Burt 2014). However, in their review of parenting literature, Taraban and Shaw (2018) found that the link between parenting and child factors is weaker compared to the influence of context‐ and parent‐related factors.

This section summarizes relevant research on ESBs concerning the child's age, gender, birth order, and negative emotionality. While research on parenting generally has supported the notion of gendered socialization of emotions (Hofmann and Doan 2018), several studies specifically on parental ESBs found no evidence of differences based on the child's gender (Baker et al. 2011; Cabecinha‐Alati et al. 2020; Dixon‐Gordon et al. 2020; Lunkenheimer et al. 2007; McKee et al. 2022; Scott and Hakim‐Larson 2021). Regarding child age, research indicates that parents adjust their socialization strategies based on their perception of children's developmental stage (Denham 2019; Morris et al. 2007), and as children grow older, parents tend to respond more dismissively (Lunkenheimer et al. 2007; McKee et al. 2022).

With respect to genetic constitution, birth order is random within a family. That is, the first and second born are, by nature and on average, no different in genetic factors regarding psychological traits. However, research shows that firstborns typically excel more in academics and careers than their siblings; a difference bound to be environmental (Baker et al. 2011; Sulloway 2007). To our knowledge, no previous studies specifically explore the relationship between birth order and parental ESBs in a population‐based sample. Research does, however, suggest that birth order affects how parents interact with their children, with firstborns less likely to be physically punished (Kim and Wang 2023), tending to spend more quality time with their parents (Price 2008), and often receiving more positive affection compared to their later‐born siblings (Bornstein 2016).

Throughout development, parents and children mutually influence each other through both bidirectional and interactional processes (Kiff et al. 2011), making it challenging to entangle causal effects. Nonetheless, child temperament represents genetically based characteristics that are visible from infancy and demonstrate stability over time (Kiff et al. 2011), making it an intriguing candidate for studying child effects. Child temperament was emphasized in Belsky's model (Belsky 1984) and has since attracted considerable attention in research on child effects. Research on parenting styles and child temperament has consistently demonstrated a small but robust association (Bornstein 2019; Taraban and Shaw 2018), with particular focus on negative emotionality, that is, psychological instability and a tendency to experience strong emotions. Children with high negative emotionality are easily aroused and challenging to soothe, which can cause significant stress for parents (Kiff et al. 2011). Studying genetic influences on parenting by analyzing a sample of adoptive parents and their children, alongside measuring the temperament of biological parents, revealed that children's negative emotionality elicits hostile parenting but does not impact parental warmth (Shewark et al. 2021). The literature on parental ESBs and children's negative emotionality is however sparse and contradictory; Scott and Hakim‐Larson (2021) found children's negative emotionality associated with mothers' rejection, however, a recent study by Thompson et al. (2020) found no association between children's negative emotionality and ESBs, challenging expectations within the field.

## 3 Knowledge Gaps in Existing Research

While research on parental ESBs has expanded, notable gaps remain (De Raeymaecker and Dhar 2022; Eisenberg 2020). First, most studies have focused on how parental ESBs influence child development, while the factors contributing to the variation in parental ESBs remain less understood. Second, with some noteworthy exceptions (e.g., Thompson et al. 2020), existing studies have tended to investigate specific factors, such as parental emotion regulation, psychopathology, or child temperament, rather than testing integrative models. Yet, it is unlikely that specific factors alone account for a substantial part of the variation, and we should systematically develop comprehensive models that illustrate how multiple factors combine to shape parenting (Bornstein 2016; Plomin 2023). Third, although parenting interventions place great importance on parents' developmental history, the significance of parents' recollections of parental ESBs within the family of origin has not been explored in conjunction with the broader perspective on predictors proposed by the process model of parental ESBs (Eisenberg et al. 1998; Eisenberg 2020). Fourth, most research has been conducted using clinical or convenience samples and relatively small sample sizes, which limits generalizability and may obscure how predictors operate in the broader population (Eisenberg 2020). Finally, fathers remain underrepresented in parenting research (Root and Denham 2010), resulting in samples that are not representative of the broader parent population.

In summary, previous research on parental ESBs has provided valuable insights but remains limited in scope, often focusing on narrow sets of predictors and relying on small or nonrepresentative samples. To our knowledge, no recent studies have examined variation in parental ESBs by combining a wide range of factors from context, parent, and child, including both parents, in a large population‐based sample. Accordingly, there is a need for comprehensive, theory‐driven research that integrates multiple determinants and provides a more representative and conceptually coherent understanding of parental ESBs.

## 4 The Current Study

The overall aim of this study was to advance understanding of variability in parental ESBs by examining a broad range of predictors across multiple domains in a large population‐based sample, including both mothers and fathers. Building on influential process models of parenting (Belsky 1984; Eisenberg et al. 1998), predictors were organized into three domains: family context (income, education, country of birth, number of children in the household, and relationship satisfaction), parent factors (gender, age, developmental history, parental stress, symptoms of anxiety and depression, alcohol use, and personality), and child factors (age, gender, birth order, and negative emotionality). Analyzing variables both collectively and separately allows an examination of their shared and unique contributions.

The study addresses two main objectives: (1) how much variance in supportive and non‐supportive ESBs is explained by each predictor domain—the parent, the context, and the child—and (2) which predictors within each domain have a specific effect on parental ESBs? Research indicates that supportive and non‐supportive ESBs represent distinct dimensions of parenting practices, so the research questions were addressed separately.

Based on Belsky's model (1984) and the subsequent review (Taraban and Shaw 2018), along with research specifically focused on parental ESBs, we expected that all three domains would predict both supportive and non‐supportive ESBs. The family context domain was expected to explain most of the variance, with higher relationship satisfaction predicting more supportive and less non‐supportive ESBs. SES was expected to contribute significantly, but mixed findings about its connection with ESBs led to caution in forming definitive hypotheses. Parent characteristics were expected to account for the second most variance, with all variables serving as significant predictors. Finally, child characteristics were expected to account for weaker but meaningful associations; with higher negative emotionality predicting less support and more non‐supportive ESBs, and increasing child age predicting greater use of non‐supportive ESBs.

## 1 Method

### 1.1 Procedures and Sample Selection

This study is part of the extensive “Parenting Practices in Norway” (PPN) research project (Bekkhus et al. 2023). Data collection took place from September to December 2022. A stratified random selection of 15,602 parents was drawn from the National Register of Citizens using an extended family‐based twin design. This number reflects the invitation pool: all parents who received invitations via email and SMS, with links to an online consent form and questionnaire. Of those invited, 5419 parents provided consent and completed all or parts of the questionnaire. Although the response rate was moderate (34.7%), it was comparable to that of other large‐scale population‐based surveys.

The PPN project's sampling strategy was parent‐based, meaning it targeted parents who were twins or related to a twin, not families with twin children. All participants were parents with at least one child aged 3–13 years and either (a) twins or siblings to twins sharing the same mother, (b) born to twins, (c) siblings born abroad, or (d) partners to any of the mentioned categories. To maximize the chances of a diverse sample, the project intentionally oversampled full‐sibling parents with cultural backgrounds from countries outside Norway. In instances of multiple children within the target age range, we requested parents to provide responses pertaining to the child closest to 8 years of age. The design intended that multiple parents could report on the same child, and if the current partner was not the biological parent, we invited both the current partner and the biological parent to participate. The study focused on parental practices, and analyses were conducted at the parent level, treating each parent's report as a separate observation. Since the study relied on parents' self‐reported practices, identifying information about the children was not collected, so it was not possible to determine the precise number of children represented. The family membership data for parents was obtained from the National Population Register, with 2115 extended families included. The twin aspect of the sample was not central to this study, but we accounted for potential dependencies arising from the sample design by including a random family‐level intercept in all models to control for family clustering, ensuring that relatedness among participants does not bias the estimates.

Of the 5419 respondents, we included parents whose data documented that the child's age fell within the desired age range of 3–13. To study relationship satisfaction, a central factor in Belsky's (1984) model, we included only those currently in a relationship. This approach ensured identical samples across regression steps and maintained theoretical coherence and methodological consistency. Applying these eligibility criteria yielded a sample of 4561 participants. Procedures for handling missing data are described in the Missing Data section and resulted in a final analytic sample of 4207 parents.

### 1.2 Ethical Considerations

The PPN project has undergone preregistration and has received approval from the Regional Committees for Medical and Health Research Ethics (reference number: REK‐2022/346141) and the Norwegian Centre for Research Data (reference number: NSD‐2022/584188). All participants provided electronic informed consent and received a universal gift card valued at 400 NOK (approximately 33 EUR) as compensation for their time.

### 1.3 Measures and Instruments

#### 1.3.1 Demographics

Parents reported their age in years, current relationship status (either in a relationship or not), education level, annual personal income, number of children in the household and country of birth. Information on parent gender was registered from the National Register of Citizens. Education was reported based on six options: no education, primary school, high school, vocational college, college or university with < 4 years of education, and college or university with more than 4 years of education. Parents reported their financial status in terms of annual personal income in increments of 100,000 NOK, ranging from ‘0’ to 2,000,000 NOK, with the final domain exceeding 2,000,000 NOK. Parents provided information for the targeted child's gender (girl, boy, other), age (year of birth) and birth order. The final sample is detailed in Table 1 in the results section.

**TABLE 1:** Descriptive statistics for sample characteristics and study instruments.

Characteristics	*n* (%) or *M* (SD)	Range
Parent gender		
Female	2460 (58.5%)	
Male	1747 (41.5%)	
Longest completed education		
Elementary or vocational college	1219 (28.9%)	
College or university < 4 years	1391 (33.1%)	
College or university > 4 years	1597 (38.0%)	
Personal income (NOK)		
Low income (< 300,000)	226 (5.4%)	
300,000–500,000	813 (19.3%)	
500,000–700,000	1672 (40.0%)	
700,000–1,000,000	965 (23.0%)	
Above 1,000,000	531 (12.6%)	
Number of children in the household		
0^a^	5 (0.1%)	
1	330 (7.8%)	
2	2261 (53.7%)	
3	1196 (28.4%)	
> 4	415 (10.0%)	
Birth country		
Norway	3849 (91.5%)	
Other	358 (8.5%)	
Child gender		
Female	2077 (49.4%)	
Male	2130 (50.6%)	
Child birth‐order		
First	1832 (43.5%)	
Second	1548 (36.8%)	
Third	674 (16.0%)	
Fourth	126 (3.0%)	
> Fifth	27 (0.7%)	
Parent age (years)	40.0 (5.2)	25–61
Child age (years)	7.4 (2.2)	3–13
Supportive ESBs	5.56 (0.98)	1–7
Non‐supportive ESBs	2.21 (0.77)	1–6
Parental stress	2.04 (0.55)	1–4.43
Anxiety & depression	1.41 (0.50)	1–4
Alcohol use	3.75 (2.03)	0–12
Extraversion	3.74 (0.77)	1–5
Agreeableness	4.11 (0.59)	1–5
Conscientiousness	3.98 (0.78)	1–5
Neuroticism	2.42 (0.81)	1–5
Openness	3.67 (0.75)	1–5
Recollected supportive ESBs	3.06 (1.51)	1–7
Recollected non‐sup. ESBs	2.99 (1.23)	1–7
Negative emotionality	3.29 (0.87)	1–5

*Note: N* = 4207. All continuous variables have complete data.

Abbreviations: Recollected non‐sup ESBs, recollected non‐supportive ESBs; *M*, mean; SD, standard deviation.

^a^ Reflects that five parents reported on a child who does not live in their household.

#### 1.3.2 Parental Emotion Socialization

ESBs were assessed using an abbreviated version of the *Coping with Children's Negative Emotions Scale* (CCNES; Fabes et al. 2001). The CCNES measures parental reactions to children's negative emotions by presenting a series of vignettes that depict children displaying negative emotions, such as “If my child falls off their bicycle and breaks it, and then becomes upset and cries, I would…”. The original CCNES instrument comprises 12 vignettes, six response domains, and 72 items. The present study used a previously validated abbreviated version of CCNES (Amundsen et al. 2023), which includes items and subscales from the original instrument to represent supportive and non‐supportive parental ESBs. The subscale Expressive Encouragement (EE; seven items), with response items like “Encourage my child to express his/her feelings of anger and frustration” was used to measure supportive ESBs. To measure non‐supportive responses, we combined items from the Minimizing Reactions (MR) scale (four items) and the Punitive Reactions (PR) scale (three items). An example of a minimizing response is “Tell my child that he/she is overreacting,” while an example of a punishing response is, “Tell my child he/she must behave, or we must go home immediately.” Parents reported the likelihood of employing specific responses to these scenarios, each response item representing a particular emotion socialization parenting strategy, rated on a 7‐point Likert scale from 1 (“very unlikely”) to 7 (“very likely”), with a higher mean score indicating more use of supportive or non‐supportive ESBs. The Cronbach's alphas were 0.84 for the supportive and 0.70 for the non‐supportive ESBs scale, respectively.

#### 1.3.3 Anxiety and Depression

*The Hopkins Symptom Checklist‐5* (HSCL‐5; Hesbacher et al. 1980), consisting of five items, was used to measure symptoms of anxiety and depression. Participants were asked to report how much they in the past 2 weeks experienced symptoms like “feeling fearful” or “feeling hopeless about the future” and rate their response on a 4‐point scale ranging from 1 (“not bothered”) to 4 (“very much bothered”), a higher mean score indicating more symptoms. The Cronbach's alpha coefficient for HSCL‐5 in the current study was 0.86.

#### 1.3.4 Alcohol Use

A short version of the *Alcohol Use Disorders Identification Test* (AUDIT‐C; Babor et al. 2001) was used to assess alcohol use. AUDIT‐C includes three items measuring the frequency of alcohol consumption: the number of units consumed during a typical drinking day, and the frequency of exceeding six or more units in a single instance. Items were responded to on 5 and 6‐point Likert scales, with a total score ranging from 0–12 points, wherein higher mean scores indicated higher alcohol use.

#### 1.3.5 Parental Stress

The *Parental Stress Scale* (PSS; Berry and Jones 1995) measures the general experience of stress and satisfaction with being a parent. Items include statements like “Caring for my child(ren) sometimes takes more time and energy than I have to give”, and respondents rate the items on a Likert scale from 1 (“strongly disagree”) to 5 (“strongly agree”), with higher scores indicating more stress. We utilized a short version consisting of seven items, which resulted in Cronbach's alpha = 0.62.

#### 1.3.6 Personality

*The Big Five Inventory‐2* (BFI‐2; Soto and John 2017) assesses five personality traits: extraversion, agreeableness, conscientiousness, neuroticism, and openness. Each item is a statement like “I am someone who tends to be quiet,” and participants report how this applies to themselves on a 5‐point rating scale, ranging from 1 (“totally disagree”) to 5 (“totally agree”), with no labels for the scale points in between, with higher mean scores indicating more of the trait. We used a form consisting of 15 items, with Cronbach's alphas estimated to 0.54 (extraversion), 0.41 (agreeableness), 0.56 (conscientiousness), 0.58 (neuroticism), and 0.44 (openness).

#### 1.3.7 Relationship Satisfaction

*Relationship Assessment Scale* (RAS; Hendrick 1988) is widely used for assessing relationship satisfaction. We administered the single‐item version, RAS‐1, which has proven to be equally as adequate as the full seven‐item instrument and is recommended to be used in large‐scale studies (Fülöp et al. 2022). The RAS‐1 item is “In general, how satisfied are you with your relationship?”, and the response is given on a 5‐point Likert scale (from 1 = not satisfied to 5 = very satisfied).

#### 1.3.8 Recollected Emotion Socialization

As a measure of parents' developmental history, we modified the CCNES described above to measure parents' recollection of their own parents' use of ESBs in the family of origin. The modification involved changing the subject of the items, for example, from “If my child falls off his/her bike and breaks it, and then gets upset and cries, I would…” to “If you fell off your bike and broke it, and then got upset and cried, your parent would…”. Apart from this modification, the instrument remained identical to the shorter version described above. This ensures that the accuracy and validity of the instrument is maintained while still allowing for the necessary adjustment. Cronbach's alpha coefficient was 0.94 for recollected supportive ESBs, and 0.84 for recollected non‐supportive ESBs.

#### 1.3.9 Children's Negative Emotionality

Children's negative emotionality was assessed using a subscale of *The Emotionality, Activity, and Sociability Temperament Survey* (EAS; Buss and Plomin 1984). Parents were asked to assess whether different statements, such as “child gets upset or sad easily,” applied to their child on a 5‐point Likert scale ranging from 1 (“very typical”) to 5 (“not at all typical”). The present study included three items from the EAS measuring negative emotionality, with a lower mean score indicating more negative emotionality. Cronbach's alpha coefficient for negative emotionality in this sample was 0.72.

### 1.4 Missing Data

After restricting the sample to parents currently in a relationship and with a child within the age range (*n* = 4561), item‐level missingness across all study variables was low (0.6%). Item‐level missingness for the outcome variables, supportive and non‐supportive ESBs, was minimal, with 0.2% missingness, and 95% of the parents completing all items.

In total, 354 participants had one or more missing values on the variables included in the regression analyses. Little's MCAR test indicated that missingness was not completely at random (*p* < 0.001), which is common in large population‐based survey data.

To evaluate whether exclusion due to missing data could introduce systematic bias, parents included in the analytic sample were compared with those with missing data on one or more variables (*n* = 354). Comparisons showed no meaningful differences in parent or child age or gender. Differences in all continuous study variables were small (Cohen's *d* < 0.10), except for alcohol use (*d* = 0.19), with higher levels among included parents. For education and household income, differences across categories were modest and graded, with included parents more likely to have higher incomes and education levels.

Given the low percentage of missing data and the large sample size, analyses were conducted using complete cases, prioritizing model simplicity, transparency, and accuracy over maximizing sample size. This resulted in a final analytical sample of 4207 parents.

### 1.5 Data Analyses

All analyses were conducted in R (version 4.4.3; R Core Team 2025), using the lme4 and lmerTest packages. As the study focused on within‐sample associations rather than population prevalence estimates, no weighting procedures were applied. Pearson's correlation coefficients were used to calculate bivariate correlations among all variables, and associations were examined to assess the degree of overlap and multicollinearity.

A hierarchical regression analysis was conducted in four steps to test the relative contributions of the three predictor groups, parent, context, and child factors, with supportive and non‐supportive ESBs analyzed separately. To account for nonindependence in data due to participants being nested within extended families (e.g., siblings, cousins, in‐laws), all regression models were estimated as multilevel models with a random intercept for family ID. Initially, a null model including only the random intercept for family was estimated to quantify the variance attributable to family‐level clustering (intraclass correlation; ICC) without predictors. Step 2 added parent characteristics (age, gender, parental stress, anxiety and depression, alcohol use, personality factors (extraversion, agreeableness, conscientiousness, neuroticism, and openness), and recollected supportive and non‐supportive ESBs). Step 3 included family context variables (education, income, relationship satisfaction, number of children in the household, and birth country). Finally, child factors (age, gender, birth order, and negative emotionality) were entered in Step 4, representing the full model. Model assumptions were inspected prior to estimation, and multicollinearity was evaluated for each regression analysis. Incremental improvements were evaluated using likelihood‐ratio tests (ANOVAs), testing whether each step contributed additional explained variance. Marginal R^2^ was estimated as an index of variance explained by fixed effects (Nakagawa and Schielzeth 2013), and ICC quantified the proportion of variance associated with family‐level clustering. The study sample size adequately meets standard conventions for the number of predictors relative to the sample size (Siddiqui 2013), but given the substantial number of predictors, a significance level of 0.01 was adopted to minimize the likelihood of Type I errors.

## 2 Results

### 2.1 Descriptives and Preliminary Analyses

Table 1 provides complete descriptions of the sociodemographic characteristics of the sample (*n* = 4207) and descriptive statistics for all study instruments. As shown in Table 2, correlations were generally small but significant, indicating the potential for predictors' unique contributions. Collinearity diagnostics were within the acceptable range (VIF = 1.01–1.79), indicating no multicollinearity (VIF < 2.5; Johnston et al. 2018).

**TABLE 2:** Bivariate correlations among study variables.

Variable	1	2	3	4	5	6	7	8	9	10	11	12	13	14	15	16	17	18	19	20	21	22
1. Supportive ESBs																						
2. Non‐sup. ESBs	−0.29**																					
3. Parent age	−0.10**	0.12**																				
4. Parent gender	−0.26**	0.30**	0.21**																			
5. Education	−0.03	−0.13**	0.18**	−0.12**																		
6. Income	−0.15**	0.13**	0.28**	0.36**	0.32**																	
7. Number of children^a^	−0.04*	0.04*	−0.02	−0.02	0.01	0.00																
8. Birth country	0.02	0.09**	0.02	0.02	0.00	−0.05*	−0.02															
9. Relationship sat.	0.08**	−0.04*	−0.09**	−0.01	−0.01	−0.03	0.06**	−0.01														
10. Parental stress	−0.11**	0.13**	−0.10**	−0.02	−0.05*	−0.09**	0.05*	0.02	−0.25**													
11. Anx. & dep.	0.03	0.00	−0.09**	−0.10**	−0.07**	−0.17**	−0.04	0.00	−0.25**	0.39**												
12. Alcohol use	−0.17**	0.15**	0.12**	0.26**	−0.01	0.21**	−0.08**	−0.14**	−0.07**	0.02	0.01											
13. Extraversion	0.16**	−0.05*	0.02	−0.11**	0.04*	0.08**	0.02	0.05*	0.12**	−0.20**	−0.22**	0.04*										
14. Agreeableness	0.34**	−0.25**	−0.07**	−0.27**	−0.03	−0.18**	0.00	0.05**	0.13**	−0.23**	−0.10**	−0.17**	0.26**									
15. Conscientiousness	0.10**	−0.07**	0.00	−0.15**	0.09**	0.07**	0.00	0.04*	0.11**	−0.20**	−0.15**	−0.14**	0.14**	0.18**								
16. Neuroticism	−0.07**	0.02	−0.11**	−0.18**	−0.04*	−0.18**	−0.03	−0.02	−0.15**	0.40**	0.50**	0.02	−0.33**	−0.26**	−0.15**							
17. Openness	0.16**	−0.07**	0.07**	0.03	0.02	0.02	0.00	0.05**	0.03	−0.07**	−0.01	0.00	0.29**	0.14**	0.01	−0.18**						
18. Rec. sup. ESB	0.26**	0.05**	−0.01	0.06**	−0.03	0.03	0.01	−0.02	0.12**	−0.15**	−0.19**	−0.01	0.15**	0.17**	0.07**	−0.17**	0.03					
19. Rec. non‐sup. ESB	0.03	0.28**	0.01	−0.01	−0.11**	−0.06**	−0.01	0.13**	−0.09**	0.17**	0.18**	0.01	−0.06**	−0.10**	−0.08**	0.13**	0.06**	−0.50**				
20. Child age	−0.06**	0.12**	0.43**	−0.02	−0.03	0.04	0.03	−0.01	0.00	−0.12**	−0.05*	0.04*	0.03	0.00	0.03	−0.07**	0.04	−0.01	0.04			
21. Child gender	0.00	0.01	−0.04*	0.01	−0.02	−0.02	0.00	0.02	0.01	0.01	0.00	−0.01	−0.01	−0.02	0.03	0.01	−0.02	0.03	−0.01	−0.04*		
22. Birth order	−0.08**	0.07**	0.42**	0.00	−0.02	0.05*	0.43**	−0.02	0.02	−0.09**	−0.06**	−0.03	0.03	0.02	0.01	−0.09**	0.02	−0.04	0.05**	0.31**	−0.04	
23. Negative em.	0.03	−0.04*	0.13**	−0.02	0.06**	0.07**	0.01	0.01	0.15**	−0.36**	−0.20**	0.00	0.13**	0.13**	0.15**	−0.24**	0.02	0.08**	−0.11**	0.18**	0.00	0.14**

Abbreviations: Anx. & depr., anxiety & depression; Negative em., negative emotionality; Non‐sup. ESBs, non‐supportive ESBs; Rec. non‐sup. ESBs, recollected non‐supportive ESBs; Rec. sup. ESBs, recollected supportive ESBs; Relationship sat., relationship satisfaction.

^a^ Number of children in the household.

* *p* < 0.01.

** *p* < 0.001.

### 2.2 Supportive ESBs Explained by Context, Parent, and Child Domains

Table 3 displays the results of the four‐step hierarchical regression for supportive ESBs. The null model showed modest clustering at the family level (ICC = 0.074). Adding parent factors explained a substantial proportion of variance (*R* ^2^_m_ = 0.27). Key predictors included parent gender, with mothers reporting higher levels of supportive ESBs than fathers. Recollected supportive and non‐supportive ESBs, along with agreeableness, openness, anxiety and depression, all predicted greater use of supportive ESBs. Conversely, parent stress levels and alcohol use were associated with less use of supportive ESBs. Family context factors only minimally increased the explained variance in Step 3 (𝚫*R* ^2^_m_ = 0.003), with more children in the household and being born outside Norway associated with reduced use of supportive ESBs. Finally, in Step 4, the inclusion of child factors slightly increased the explained variance (*R* ^2^_m_ = 0.28), with higher age and birth order predicting less use of supportive ESBs. None of the family context factors remained significant in the full model. Comparing the four models using Analysis of Variance revealed that all steps showed a significant increase in explained variance, with Steps 2 and 4 accounting for the majority of the variance.

**TABLE 3:** Hierarchical regression results for supportive and non‐supportive ESBs.

Variable	Supportive ESB					Non‐supportive ESB				
	*β*	SE *β*	*R* ^2^_m_	𝚫 *R* ^2^_m_	ICC	*β*	SE *β*	*R* ^2^_m_	𝚫 *R* ^2^_m_	ICC
Step 1 null model^a^					0.074					0.107
Constant	0.01	0.02				0.00	0.02			
Step 2 parent factors			0.268	0.268**	0.092			0.267	0.267**	0.134
Constant	0.55	0.04				−0.66	0.04			
Parent age	−0.04*	0.01				0.07**	0.01			
Parent gender	−0.39**	0.03				0.47**	0.03			
Parental stress	−0.07**	0.02				0.10**	0.02			
Anx. & depr.	0.10**	0.02				−0.03	0.02			
Alcohol use	−0.09**	0.01				0.05**	0.01			
Extraversion	0.02	0.02				0.03	0.01			
Agreeableness	0.20**	0.01				−0.17**	0.01			
Conscientiousness	0.01	0.01				0.02	0.01			
Neuroticism	−0.02	0.02				0.00	0.02			
Openness	0.10**	0.01				−0.09**	0.01			
Rec. supportive ESB	0.35**	0.02				0.27**	0.02			
Rec. non‐sup. ESB	0.22**	0.02				0.39**	0.02			
Step 3 family context			0.271	0.003*	0.090			0.278	0.011**	0.121
Constant	0.54	0.05				−0.59	0.05			
Parent age	−0.03	0.01				0.07**	0.01			
Parent gender	−0.37**	0.03				0.41**	0.03			
Parental stress	−0.07**	0.02				0.09**	0.02			
Anx. & depr.	0.10**	0.02				−0.03	0.02			
Alcohol use	−0.09**	0.01				0.06**	0.01			
Extraversion	0.02	0.02				0.03	0.01			
Agreeableness	0.20**	0.02				−0.17**	0.01			
Conscientiousness	0.01	0.01				0.02	0.01			
Neuroticism	−0.02	0.02				0.00	0.02			
Openness	0.10**	0.01				−0.09**	0.01			
Rec. supportive ESBs	0.35**	0.02				0.26**	0.02			
Rec. non‐sup. ESBs	0.22**	0.02				0.38**	0.02			
Education	0.00	0.01				−0.08**	0.01			
Income	−0.02	0.02				0.04**	0.02			
Rel. satisfaction	0.02	0.01				0.00	0.01			
Number of children	−0.05**	0.01				0.05**	0.01			
Birth country	−0.10**	0.05				0.19**	0.05			
Step 4 child factors			0.278	0.007**	0.086			0.287	0.009**	0.118
Constant	0.60	0.06				−0.63	0.06			
Parent age	0.03	0.02				0.02	0.02			
Parent gender	−0.40**	0.03				0.44**	0.03			
Parental stress	−0.08**	0.02				0.10**	0.02			
Anx. & depr.	0.10**	0.02				−0.03	0.02			
Alcohol use	−0.09**	0.01				0.06**	0.01			
Extraversion	0.02	0.02				0.03	0.01			
Agreeableness	0.20**	0.02				−0.16**	0.01			
Conscientiousness	0.02	0.01				0.02	0.01			
Neuroticism	−0.03	0.02				0.00	0.02			
Openness	0.10**	0.01				−0.09**	0.01			
Rec. supportive ESBs	0.34**	0.02				0.26**	0.02			
Rec. non‐sup. ESBs	0.23**	0.02				0.37**	0.02			
Education	−0.01	0.02				−0.07**	0.01			
Income	−0.02	0.02				0.05**	0.02			
Rel. satisfaction	0.02	0.01				0.00	0.01			
Number of children	−0.01	0.02				0.04**	0.02			
Birth country	−0.11**	0.05				0.20**	0.05			
Child age	−0.06**	0.02				0.11**	0.02			
Child gender	−0.01	0.03				0.00	0.03			
Birth order	−0.07**	0.02				0.00	0.02			
Negative emotionality	−0.01	0.01				0.00	0.01			

*Note: N* = 4207 for all models.

Abbreviations: Anx. & Depr., anxiety & depression; ICC, intraclass correlation coefficient, that is, the proportion of variance explained by the family level random effects; *R* ^2^_m_, marginal *R* ^2^; Rec. non‐sup. ESBs, recollected non‐supportive ESB; Rec. supportive ESBs, recollected supportive ESB; Rel. satisfaction., relationship satisfaction.

^a^ Including only a random intercept accounting for family‐level clustering.

* *p* < 0.01.

** *p* < 0.001.

### 2.3 Non‐Supportive ESBs Explained by Context, Parent, and Child Domains

For non‐supportive ESBs, the null model likewise indicated modest family‐level clustering (ICC = 0.107). In Step 2, parent factors again accounted for a large proportion of variance (*R* ^2^_m_ = 0.27), with parent gender as a strong predictor, indicating that fathers reported greater use of non‐supportive ESBs than mothers. The main predictors, aside from gender, were recollected ESBs, with both supportive and non‐supportive recollected ESBs being notably influential. Higher levels of stress and alcohol consumption predicted greater use of non‐supportive ESBs, while symptoms of anxiety and depression, along with stronger traits of agreeableness and openness, were associated with decreased use of non‐supportive ESBs.

Adding family context factors in Step 3 yielded a small but significant increase in (*R* ^2^_m_ to 0.28, 𝚫*R* ^2^_m_ = 0.011). Higher education predicted less use of non‐supportive ESBs, while higher income, an increasing number of children in the family, and a birth country other than Norway predicted greater use of non‐supportive ESBs. The complete model in Step 4 added child factors, resulting in a small but significant increase in explained variance (*R* ^2^_m_ = 0.29, 𝚫*R* ^2^_m_ = 0.009), with child age as the only significant predictor, indicating that increasing age is associated with greater use of non‐supportive ESBs.

To examine whether the overall pattern of associations was consistent across parent gender, we reestimated the models separately for mothers and fathers (Tables S1–S3). The results mirrored the main findings: parent factors remained the strongest predictors of both supportive and non‐supportive ESBs, while contextual and child domains added only modest explanatory power. Although some coefficients varied in strength between mothers and fathers, the overall pattern of predictors remained mostly consistent, supporting the robustness of the main findings.

## 3 Discussion

This study is the first to employ a population‐based sample to investigate the contributions of predictors across three domains—family context, parent characteristics, and child characteristics—to parental ESBs, including both mothers and fathers. Collectively, the three domains explained 28% of the variance in supportive ESBs and 29% in non‐supportive ESBs. All three domains made significant contributions, but as a novelty, the results distinctly underscored parent factors, specifically parent gender and recollected ESBs from the family of origin, as the most influential predictors. Additionally, other parent factors, including parenting stress, alcohol use, and the personality traits agreeableness and openness, demonstrated unique effects on both supportive and non‐supportive ESBs. In contrast, symptoms of anxiety and depression were only related to supportive ESBs. Contextual factors, including income, education, the number of children in the family, and parents' birth country, predicted non‐supportive ESBs but showed no relation to supportive ESBs. Child age and birth order had unique effects on supportive ESBs, while only child age demonstrated effects on non‐supportive ESBs. The correlation between supportive and non‐supportive ESBs (*r* = 0.29) supports the notion that they are related yet distinct constructs.

The modest ICCs indicate some similarities among extended family members and support the interpretation that most variance emerges at the individual‐parent level. This finding aligns with the strong predictive power of parents' recollected ESBs, consistent with theory and empirical evidence showing that developmental history shapes parenting (Belsky 1984; Buston et al. 2022) and that emotion‐related parenting practices show intergenerational continuity. Taken together, these findings could suggest that parental ESBs are shaped less by extended family context and more by internalized recollections of caregiving.

### 3.1 Parent Characteristics as the Main Predictors of ESBs

Finding that parent characteristics account for most of the variation in parental ESBs aligns with Belsky's original theory, which emphasizes parent factors as the main predictors for parenting. This finding contrasts with Taraban and Shaw's (2018) revision, which highlighted contextual factors as the empirically dominant predictors. While parenting undoubtedly emerges from a complex interplay between intrinsic parent factors and contextual conditions (Eisenberg et al. 1998), the present results suggest that parents' gender and psychological dispositions exert substantial and direct influences on parenting, potentially mediating how external context translates into parental ESBs. From a developmental psychological perspective in which parental ESBs are shaped through a life course process, these findings indicate that parental ESBs reflect caregiving experiences in the family of origin and enduring personality traits rather than being primarily determined by current contextual or child‐related factors.

We found that mothers reported to engage in more supportive and less non‐supportive ESBs, compared to fathers. Although the observed gender differences are consistent with prior research on ESBs (Baker et al. 2011; Cassano et al. 2014; Gottman et al. 1996; McKee et al. 2022; Nelson et al. 2009; Wong et al. 2009), the gender differences are surprisingly pronounced given Norway's ranking as the second most gender‐equal country globally (World Economic Forum, n.d.). Parenting practices are embedded in culture and time, and there has been a marked increase in the engagement of Norwegian fathers in childcare over recent years (Egeland 2021), with 65% of fathers utilizing 15 weeks of full parental leave (Statistics Norway 2024). This trend could suggest a convergence in parenting strategies between mothers and fathers. Supporting this notion, a Swedish study examining three cohorts (1958, 1981, and 2011) revealed that while mothers are still viewed as the most supportive parent, there has been a shift, with an increasing percentage of individuals reporting that they turn to both fathers and mothers when feeling distressed (Trifan et al. 2014). Nonetheless, this study's findings on gender differences are strengthened by their alignment with similar findings in a recent Norwegian study (Bjørk et al. 2024), indicating the existence of robust gender differences in parental ESBs even in societies with high levels of gender equality. This suggests that more studies need to investigate the mechanisms that might explain these differences, including genetic, contextual, historical, and cultural factors.

Recollected supportive ESBs predicted current supportive ESBs, and similarly, the recollected non‐supportive parental strategies positively predicted non‐supportive ESBs, indicating a continuity of parenting practice across generations on a group level. This continuity between generations can be attributed to various mechanisms. One potential mechanism of direct effect occurs when genetically heritable traits are passed on, predisposing the subsequent generation to specific environments or behavior. This phenomenon is known as passive gene–environment correlations (Kerr and Capaldi 2019). Genetically informed studies of parenting have found evidence of heritable components in parenting practices (Bornstein 2016; Kretschmer 2023; McGuire et al. 2012), and two meta‐analyses reveal the parental warmth dimension to show heritability estimates between 30% and 40% (Kendler and Baker 2007; Klahr and Burt 2014). Although genetic heritability has not yet been documented in parental emotion socialization research, it presents a plausible explanation for parts of the observed association, given that ESBs are related to broader parenting dimensions (Eisenberg 2020).

Another example of direct effect is proposed by social learning theory (Patterson 2016) and Morris et al.'s (2007) tripartite model of emotion regulation, which emphasizes that parenting behavior is shaped via observational learning, modeling, and social reinforcement. Attachment theory offers further insight into the mechanisms of social learning by explaining how interactions with caregivers lead children to develop inner working models, which subsequently will shape their approach to parenting in adulthood (Cao et al. 2023; Leerkes and Siepak 2006). The mechanisms involved in intergenerational transmission are not entirely understood, although it seems plausible that biological, social, and psychological factors contribute to explaining this transmission (Kerr and Capaldi 2019; Rothenberg 2019).

The moderate effect sizes between recollected and current ESBs indicate that while parenting practices are transmitted across generations, there is a significant divergence where parents modify or depart from the methods they recollected from their upbringing. Some parents may intentionally treat their children differently from their own experiences, aiming to break negative social heritability or pass down values and practices distinct from those of their parents, such as being encouraging instead of punitive towards the expression of anger or sadness. In their study of different cohorts of Swedish parents, Trifan et al. (2014) found evidence of a marked decline from 1981 to 2011 in directive control and punitive practices and that parents increasingly allowed children to express anger towards the parents (Trifan et al. 2014). A potential underlining mechanism was explored in a study by Milan et al. (2021), examining mothers with and without a history of child maltreatment; they found that greater use of cognitive processing language in describing childhood narratives was associated with reduced intergenerational similarity of non‐supportive ESBs, suggesting that mechanisms such as mentalization play a role in the transmission.

When both recalled supportive and recalled non‐supportive ESBs were included in the regression model, recalled supportive ESBs became a substantial predictor of non‐supportive ESBs, and recollected non‐supportive ESBs became a substantial predictor of the use of supportive ESBs, despite showing no relation in the correlation analyses (Table 2). This finding suggests classical suppression effects, where the inclusion of a predictor variable reduces the error in another predictor, which becomes a significant predictor despite having no correlation with the outcome variable (Gaylord‐Harden et al. 2010). The findings suggest that given, recollection of the same degree of support towards negative emotions, recalling more punishment and minimizing predicts practicing more supportive ESBs towards one's own child. This finding is supported by studies documenting that parents with adverse experiences with caregivers may desire to break the cycle of negative parenting by intentionally shifting their strategy to be more supportive towards their child (Tadjine and Swords 2024). The additional suppressor effect, that is, finding that controlling for recollected punishment and minimizing, remembering more supportive ESBs predict greater use of punishment and minimizing, however, appears counterintuitive and necessitates careful interpretation. One explanation may be that the overall amount of emotional responses the parents recall (positive or negative) predicts higher levels of ESBs, regardless of their valence. A person‐centered approach could be beneficial for further exploration (Morin et al. 2017).

Interestingly, heightened symptoms of anxiety and depression predicted increased use of supportive ESBs after controlling for other factors (i.e., also consistent with a suppressor effect), and no association with non‐supportive ESBs. This result contrasts with the established literature, which consistently finds that parents experiencing anxiety and depression engage in more non‐supportive responses (Breaux et al. 2022; Godleski et al. 2020). This unexpected positive association between anxiety and depression symptoms and supportive ESBs, coupled with the absence of a link to non‐supportive ESBs, merits further exploration. One potential explanation may lie in the population‐based nature of the sample, as opposed to the clinical samples predominantly used in previous studies. In this context, anxiety and depression symptoms largely reflect subclinical variation and pronounced floor effects, rather than clinically elevated symptomatology. Within such nonclinical populations, the report of anxiety and depression symptoms could reflect greater emotional awareness and sensitivity, rather than a clinical disorder, which may facilitate constructive engagement with their children's negative emotions, thereby fostering supportive interactions. These findings invite a reevaluation of assumptions derived from clinical studies of adult psychopathology and suggest avenues for future research into the nuances of emotional socialization in diverse populations.

In line with previous studies (Amundsen et al. 2023; Godleski et al. 2020), we found that parents who used more alcohol also used more non‐supportive and less supportive ESBs. Alcohol use may interfere with the cognitive and emotional ability to mentalize and hinder the ability to regulate emotions effectively by affecting specific brain regions responsible for emotion regulation (Hajal and Paley 2020). Effective emotion regulation skills are, in turn, a crucial component for parents to optimally practice ESBs (Eisenberg 2020; Hajal and Paley 2020).

Consistent with previous research (Nelson et al. 2023), parents experiencing higher levels of parental stress reported less supportive and more non‐supportive reactions when children displayed negative emotions. Nelson et al. (2023) explains the relationship between non‐supportive ESBs and parenting stress as a negative cycle, where non‐supportive ESBs result in less regulated children, which, in turn, contributes to parenting stress. However, it may also be that poorly regulated children elicit both more stress and, thus, more non‐supportive parenting from the parent (Kiff et al. 2011). As shown in Table 2 in the results, parenting stress, alcohol use, and anxiety and depression are related to other factors in an intricate pattern, and understanding the mechanisms requires in‐depth analyses in future research.

Supporting Belsky's (1984) and Eisenberg et al.'s (1998) models, and corroborating previous research (Prinzie et al. 2009), personality factors were significantly linked to ESBs. Consistent with Hughes and Gullones' (2010) meta‐analyses, agreeableness and openness emerged as prominent predictors and showed the same pattern: higher levels of these traits predicted more supportive ESBs and fewer non‐supportive ESBs. The strongest association was observed with agreeableness. As agreableness reflects parents' interpersonal orientation on a continuum from antagonism to empathy, one interpretation is that the finding underscores the importance of empathy in effectively responding to children's negative emotions. It should be noted that the internal consistency of the Big Five scales was low to moderate, and associations involving personality traits should therefore be interpreted with some caution. However, low reliability due to few items in Big Five primarily attenuates observed effects (e.g., Thalmayer et al. 2011), suggesting that the observed associations are conservative estimates rather than false positives.

### 3.2 Family Context Effects

Our findings suggest that higher education might discourage minimizing and punitive responses to children's negative emotions, and that parents with higher income can be more inclined to use non‐supportive ESBs. However, neither education nor income demonstrated unique effects on supportive ESBs, nuancing findings from previous studies linking higher SES to more supportive and less non‐supportive parenting (Lugo‐Candelas et al. 2015; Shaffer et al. 2012) and contrasting findings that higher education was associated with a lower likelihood of having an emotion‐supportive profile (McKee et al. 2022). Taraban and Shaw (2018) observed that the effects of contextual factors are more pronounced in low‐SES groups than in middle‐ or upper‐class samples, possibly due to differences in variability, which might explain the divergent effects in this sample. Additionally, in Norway, income and education are not as correlated as in other countries, which may help explain why these two factors predict opposite effects on non‐supportive ESBs in this sample. These insights challenge the view of SES as a uniform predictor of parenting behaviors and compel us to explore how different components of SES might uniquely influence parental strategies. One explanation is that families with children in Norway exist within a context characterized by robust social welfare structures and comparatively modest class disparities. Welfare benefits like universal access to government‐regulated, high‐quality, and subsidized kindergartens might compensate for some SES differences, potentially making low SES less detrimental for parental practices compared to countries with less government support. This argument is strengthened by a similar finding in a recent Norwegian study (Bjørk et al. 2024). Future studies replicating these measures in countries with more socioeconomic disparities may contribute to understanding the nuances in the role of SES for parenting practices.

We found that parents born in Norway used less minimizing and punishing ESBs compared to parents born in other countries. This finding is expected, as cooperation with the child is highly valued in Norwegian culture, and exemplary child‐rearing is defined by a child‐centric and dialogue‐oriented approach (Hollekim 2016). The finding aligns with robust research findings implying that parents in Western cultures tend to use less non‐supportive ESBs but also more supportive ESBs (for a review, see Raval and Walker 2019). However, it should be noted that sharing birth country does not necessarily mean sharing the same culture, as acculturation levels can vary widely among multicultural families and individuals. Parents with different cultural backgrounds may have adopted Norwegian cultural norms and behaviors to varying degrees. Furthermore, the CCNES instrument was created within a Western context, meaning that the definitions of supportive and non‐supportive behaviors are framed by Western cultural norms.

### 3.3 Child Effects

Parental ESBs differed based on children's age and birth order, but were not related to child gender or negative emotionality, neither in the correlation nor regression analyses. The lack of prediction by children's gender is consistent with previous research and implies the robustness of these findings (Bjørk et al. 2024; Endendijk et al. 2016; McKee et al. 2022; Thompson et al. 2020). The findings suggest that parents employ similar emotion socialization strategies for boys' and girls' expressions of negative emotions. However, the absence of an observed relationship both in this and earlier research may be attributed to the assessment of negative emotions as a global construct. Several studies have identified effects of children's gender when examining discrete emotions, rather than general emotions (Chaplin et al. 2010; Garside and Klimes‐Dougan 2002; Root and Denham 2010). Consequently, while findings indicate that parents overall respond similarly to the negative emotions of sons and daughters, it is possible that child‐driven gender effects related to discrete emotions exist but are masked when measuring negative emotions as a global construct.

Also aligning with previous studies (Lunkenheimer et al. 2007; McKee et al. 2022), parents report using less supportive ESBs and more non‐supportive ESBs as children age. The tendency to provide more support for younger children might respond to a developmental need, and some studies have found that ESBs strategies may have different effects on child outcomes as a function of children's age (Denham 2019).

Results showed that parents used less supportive ESBs towards children born later in the sibling order. This finding can be explained by dilution theory (Price 2008), which suggests that parental resources are finite and decrease with each child born. Assuming that behaving supportively in the face of a child's negative emotion is demanding for the parent (Kiff et al. 2011), dilution theory provides a compelling explanation for this finding. With subsequent children, the strain on parental resources may reduce parents' ability to provide consistent emotional support, contributing to a shift towards more non‐supportive behavior.

Finding no effect of children's negative emotionality is noteworthy, as previous research has found a consistent link between higher levels of negative emotionality and harsh parenting (Taraban and Shaw 2018), and negative emotionality represents more strain on the parent (Kiff et al. 2011). However, this finding is supported by results from a previous study on ESBs (Thompson et al. 2020) and suggests that parental sensitivity and responsiveness to a child's emotional cues may not be considerably affected by the intensity or frequency of the child's negative emotionality. It might also be that the mechanism in a population‐based sample is different from clinical samples, where parents' non‐supportive reactions and children's negative emotions can escalate into vicious coercive cycles (Patterson 2016). Perhaps most parents adjust and cope with their child's heightened negative emotionality, with this negative coercive cycle being more pronounced only in clinical or risk samples.

## 4 Strengths and Limitations

The present study expands the existing literature by examining a wide range of factors grounded in influential parenting process models (Belsky 1984; Eisenberg 2020) providing a comprehensive overview of potential determinants of parental ESBs. As a novel contribution, the study incorporates the recollection of parental emotion socialization from the family background. The findings are strengthened by a large, population‐based sample including both mothers and fathers.

The study had several noteworthy limitations that should be considered when interpreting the results. First, due to its cross‐sectional nature, the findings are purely descriptive, meaning that observed associations, such as between parental characteristics and ESBs, should be interpreted as correlational patterns rather than developmental mechanisms. For example, while parental stress and personality predicted both supportive and non‐supportive ESBs, it cannot be determined whether these factors shape parenting or arise as consequences of the same family dynamics.

Second, the weak associations between child factors and ESBs may partly reflect methodological constraints. The relatively large span in child ages might mask nonlinear associations or developmental differences in how children's characteristics relate to ESBs. In addition, the cross‐sectional design limits the ability to capture changes or bidirectional processes over time. Longitudinal designs are needed to better clarify these issues.

Third, data rely on self‐reports, and parents' descriptions of their own behavior might vary with self‐awareness and due to the social desirability effect, possibly leading to an underestimation of the links with negatively valued behaviors such as non‐supportive ESBs and alcohol use (Paulhus 1984). At the same time, self‐perceptions offer insight into parents' subjective understanding of their emotional responses, an important aspect of emotion socialization in itself.

Fourth, the study's relatively narrow operationalization of supportive ESBs provided a measure closely aligned with Gottman's conceptualization of supportive ESBs. While this strengthens internal validity, it may have captured a more specific aspect of ESBs and limited the comparability with studies that have used a broader measure of supportive ESBs. Fifth, small effect sizes indicating relationships among factors—that is, parental stress, alcohol use, children's birth order, and ESBs—while present, may not have substantial practical implications. Findings indicate that ESBs are influenced by many interconnected factors, so the results should be viewed as reflecting probabilistic trends.

Sixth, some aspects of the sample composition may limit its representativeness and the generalizability of the findings. Compared to the national parent population, the sample included a higher proportion of parents with higher education, fewer parents from minority backgrounds, and analyses were restricted to parents who were currently in a relationship. These characteristics, together with stress‐ and motivation‐related factors that might also have affected participant selection, should be considered when interpreting associations between ESBs and, for instance, SES, parental stress, and alcohol use. For anxiety and depression, limited variability in symptom levels and floor effects in a population‐based sample, rather than selective recruitment, are likely the primary methodological considerations, which may alter or attenuate associations with emotion socialization behavior. In addition, parents who were twins or born to twins were oversampled. Although twins are generally comparable to non‐twins in terms of psychological and demographic characteristics (e.g., Barnes and Boutwell 2013; Mönkediek et al. 2020), this design feature may nonetheless introduce subtle selection biases. Furthermore, in a Nordic welfare system with relatively small socioeconomic differences and universal childcare, the predictive value of education and income on ESBs might be attenuated, thereby amplifying the dominance of parental rather than contextual factors. Together, these factors warrant caution when generalizing the findings; however, the use of a large, population‐based sample remains a major methodological strength of the study.

Furthermore, the country of birth served as a proxy for culture, and along with key instruments and theories developed in a Western context, this can potentially skew results and obscure significant cultural nuances. Lastly, the selection and categorization of predictors may influence the relative strength of the three domains. For example, parents' recollections of ESBs in their family of origin could be conceptualized as representing family context rather than a parental factor, potentially altering the relative strength of the three domains observed in this study.

### 4.1 Implications and Future Research

Highlighting parent factors as the primary influence on parental ESBs can strengthen the case for parenting interventions that help parents understand how their unique experiences and characteristics shape their parenting, thereby identifying strategies to foster adaptive emotional interactions with their children. This perspective resonates with the emerging field of “personalized medicine” (Goetz and Schork 2018), suggesting a parallel conceptual approach of “personalized parenting support”. In this framework, interventions are tailored to parents' specific emotional and psychological profiles and situations, rather than relying on uniform strategies. For instance, interventions could focus on cultivating empathy and perspective‐taking for parents low in agreeableness and openness, emphasize emotion regulation skills for parents experiencing high parental stress, or address socioeconomic stressors when these represent key contextual challenges.

The findings encourage the further exploration of psychological aspects of the parent, specifically individuals' unique subjective perceptions of past and present experiences, and how gender may mediate these effects. To increase the ecological validity of future findings, researchers should increasingly incorporate observational methods, third‐party assessments, and qualitative as well as cross‐cultural studies into research designs. Moreover, future research could explore other relevant predictors, such as trauma history and various forms of social support, while also considering the extended family, including partners and in‐laws, and examining family dynamics. Additionally, understanding the underlying mechanisms, such as the role of genetic predispositions and their expression in different environmental conditions, remains an important area for further exploration. Future research could also benefit from multivariate approaches, such as SEM, to examine supportive and non‐supportive ESBs simultaneously and capture shared as well as distinct predictors.

## 5 Conclusions

Overall, the study provided significant new insights into the association between parental ESBs and a broad range of factors, emphasizing the importance of parental characteristics, especially gender and recollected ESBs from the family of origin. Given the complex nature of parenting, small effect sizes are not to be understood as trivial; rather, they signify the need to patiently build nuanced models that capture how multiple factors combine (Bornstein 2016; Plomin 2023). Achieving a complete understanding is unlikely, but by using a large‐scale sample, the study provides nuanced, holistic knowledge within a particular cultural context. The findings advance theoretical understanding of parental emotion socialization and provide knowledge that can guide practical efforts to support parents in promoting children's social and emotional development.

## Author Contributions

**Yvonne Severinsen:** conceptualization, methodology, investigation, data curation, formal analysis, visualization, writing – original draft preparation, writing – review and editing. **Rune Flaaten Bjørk:** conceptualization, methodology, funding acquisition, supervision, writing – review and editing. **Nikolai Haahjem Eftedal:** methodology, data curation, formal analysis, visualization, supervision, writing – review and editing. **Eivind Ystrom:** conceptualization, methodology, funding acquisition, data curation, formal analysis, supervision, writing – review and editing. **Egil Nygaard:** conceptualization, methodology, funding acquisition, project administration, investigation, data curation, writing – review and editing. **Stella Tsotsi:** formal analysis, writing – review and editing. **Karine Maria Porpino Viana:** conceptualization, methodology, funding acquisition, project administration, writing – review and editing. **Mona Bekkhus:** conceptualization, methodology, funding acquisition, project administration, writing – review and editing. **Evalill Bølstad:** methodology, supervision. Evalill Bølstad passed away during manuscript preparation and was unable to approve the final draft, all remaining authors accept responsibility for the final content.

## Funding

This work was supported by the Norwegian Directorate for Children, Youth and Family Affairs (Bufdir; grant number 2020/54751‐4), the Norwegian Research Council (grant number 288083), and the University of Oslo. Eivind Ystrøm is supported by the European Union (GeoGen 101045526, ESSGN 101073237), the Swedish Research Council (2024‐06499_VR), and the Research Council of Norway (336078, 288083, 331640).

## Conflicts of Interest

The authors declare no conflicts of interest.

## Supporting information


**Data S1:** sjop70082‐sup‐0001‐DataS1.docx.

## Data Availability

The data, materials, and codes required to reproduce the analyses presented are available from the corresponding author upon request and upon ethical approval. The analyses presented here were not preregistered; however, preregistration for the main project is available at https://osf.io/k7ude/.

## References

[sjop70082-bib-0001] Amundsen, V. R. , A. Gjesmoe , E. Andreassen , et al. 2023. “Alcohol Use and Parental Socialization of Emotion in a Population‐Based Sample.” Parenting: 1–38. 10.1080/15295192.2025.2465988.37346458

[sjop70082-bib-0002] Avinun, R. , and A. Knafo . 2014. “Parenting as a Reaction Evoked by Children's Genotype: A Meta‐Analysis of Children‐As‐Twins Studies.” Personality and Social Psychology Review 18, no. 1: 87–102. 10.1177/1088868313498308. 23940232

[sjop70082-bib-0003] Babor, T. F. , J. Higgins‐Biddle , J. B. Saunders , and M. Monteiro . 2001. “AUDIT‐The Alcohol Use Disorders Identification Test: Guidelines for Use in Primary Heath Care. Second Edition.” WHO, 1–40.

[sjop70082-bib-0004] Baker, J. K. , R. M. Fenning , and K. A. Crnic . 2011. “Emotion Socialization by Mothers and Fathers: Coherence Among Behaviors and Associations With Parent Attitudes and Children's Social Competence.” Social Development 20, no. 2: 412–430. 10.1111/j.1467-9507.2010.00585.x. 21532915 PMC3082208

[sjop70082-bib-0005] Barnes, J. C. , and B. B. Boutwell . 2013. “A Demonstration of the Generalizability of Twin‐Based Research on Antisocial Behavior.” Behavior Genetics 43, no. 2: 120–131. 10.1007/s10519-012-9580-8. 23274656 PMC3683969

[sjop70082-bib-0006] Bekkhus, M. , R. F. Bjork , K. M. P. Viana , et al. 2023. “Parenting Practice in Norway—A Mixed Methods Sequential Explanatory Study of Emotion Socialization.” OSF. https://osf.io/k7ude/.

[sjop70082-bib-0007] Belsky, J. 1984. “The Determinants of Parenting: A Process Model.” Child Development 55, no. 1: 83–96. 10.2307/1129836. 6705636

[sjop70082-bib-0008] Belsky, J. , R. Conger , and D. M. Capaldi . 2009. “The Intergenerational Transmission of Parenting: Introduction to the Special Section.” Developmental Psychology 45, no. 5: 1201–1224. 10.1037/a0016245. 19702385

[sjop70082-bib-0009] Berry, J. O. , and W. H. Jones . 1995. “The Parental Stress Scale: Initial Psychometric Evidence.” Journal of Social and Personal Relationships 12, no. 3: 463–472. 10.1177/0265407595123009.

[sjop70082-bib-0010] Bjørk, R. F. , S. S. Havighurst , E. Fredriksen , and E. Bølstad . 2024. “Up You Get: Norwegian Parents' Reactions to Children's Negative Emotions.” Scandinavian Journal of Psychology 65: 1039–1054. 10.1111/sjop.13051. 38952033

[sjop70082-bib-0011] Bornstein, M. H. 2016. “Determinants of Parenting.” In Developmental Psychopathology, 1–91. John Wiley & Sons, Ltd. 10.1002/9781119125556.devpsy405.

[sjop70082-bib-0092] Bornstein, M. H. , ed. 2019. Handbook of Parenting: Volume I: Children and Parenting, Third Edition. 3rd ed. Routledge. 10.4324/9780429440847.

[sjop70082-bib-0012] Breaux, R. P. , J. Lewis , A. R. Cash , D. M. Shroff , K. L. Burkhouse , and A. Kujawa . 2022. “Parent Emotion Socialization and Positive Emotions in Child and Adolescent Clinical Samples: A Systematic Review and Call to Action.” Clinical Child and Family Psychology Review 25, no. 1: 204–221. 10.1007/s10567-022-00388-2. 35201539

[sjop70082-bib-0013] Buss, A. H. , and R. Plomin . 1984. Temperament: Early Developing Personality Traits. Lawrence Earlbaum.

[sjop70082-bib-0093] Buston, K. , R. O’Brien , and K. Maxwell . 2022. “The Case for Targeted Parenting Interventions with Reference to Intergenerational Transmission of Parenting: Qualitative Evidence from Three Studies of Marginalised Mothers’ and Fathers’ Participation in Parenting Programmes.” Child Care in Practice 28, no. 3: 274–289. 10.1080/13575279.2020.1812533. 35663503 PMC7612789

[sjop70082-bib-0014] Cabecinha‐Alati, S. , H. Malikin , and T. C. Montreuil . 2020. “Emotion Regulation and Personality as Predictors of Mothers' Emotion Socialization Practices.” Family Relations 69, no. 5: 1055–1072. 10.1111/fare.12501.

[sjop70082-bib-0015] Cao, H. , N. Zhou , and E. M. Leerkes . 2023. “Primiparous Mothers' Parenting Self‐Efficacy in Managing Toddler Distress: Childhood Nonsupportive Emotion Socialization, Adult Attachment Style, and Toddler Temperament as Antecedents.” Emotion 23: 2205–2218. 10.1037/emo0001233. 36931841 PMC10504413

[sjop70082-bib-0094] Capaldi, D. M. , K. C. Pears , D. C. R. Kerr , and L. Owen . 2008. “Intergenerational and Partner Influences on Fathers’ Negative Discipline.” Journal of Abnormal Child Psychology 36: 347–358. 10.1007/s10802-007-9182-8. 17899359 PMC2394194

[sjop70082-bib-0016] Cassano, M. , J. Zeman , and W. Sanders . 2014. “Responses to Children's Sadness: Mothers' and Fathers' Unique Contributions and Perceptions.” Merrill‐Palmer Quarterly 60: 1–23. 10.1353/mpq.2014.0004.

[sjop70082-bib-0095] Chaplin, T. M. , J. Casey , R. Sinha , and L. C. Mayes . 2010. “Differences in Caregiver Emotion Socialization of Low‐Income Toddlers.” New Directions for Child and Adolescent Development 128: 11–27. 10.1002/cd.266. PMC2917975 20552657

[sjop70082-bib-0017] Conger, R. D. , J. Belsky , and D. M. Capaldi . 2009. “The Intergenerational Transmission of Parenting: Closing Comments for the Special Section.” Developmental Psychology 45, no. 5: 1276–1283. 10.1037/a0016911. 19702391

[sjop70082-bib-0018] Conger, R. D. , T. J. Schofield , and T. K. Neppl . 2013. “Disrupting Intergenerational Continuity in Harsh and Abusive Parenting: The Importance of a Nurturing Relationship With a Romantic Partner.” Journal of Adolescent Health 53, no. 4 Suppl: S11–S17. 10.1016/j.jadohealth.2013.03.014. PMC5054751 24059934

[sjop70082-bib-0019] De Raeymaecker, K. , and M. Dhar . 2022. “The Influence of Parents on Emotion Regulation in Middle Childhood: A Systematic Review.” Children 9, no. 8: 1200. 10.3390/children9081200. 36010090 PMC9406957

[sjop70082-bib-0020] Denham, S. A. 2019. “Emotional Competence During Childhood and Adolescence.” In Handbook of Emotional Development, edited by V. LoBue , K. Pérez‐Edgar , and K. A. Buss , 493–541. Springer International Publishing. 10.1007/978-3-030-17332-6_20.

[sjop70082-bib-0021] Dixon‐Gordon, K. L. , N. P. Marsh , K. E. Balda , and J. D. McQuade . 2020. “Parent Emotion Socialization and Child Emotional Vulnerability as Predictors of Borderline Personality Features.” Research on Child and Adolescent Psychopathology 48, no. 1: 135–147. 10.1007/s10802-019-00579-6. 31388862

[sjop70082-bib-0022] Egeland, C. 2021. “Barnefamilienes Hverdagsliv i Norge 2021.”

[sjop70082-bib-0023] Eisenberg, N. 2020. “Findings, Issues, and New Directions for Research on Emotion Socialization.” Developmental Psychology 56, no. 3: 664–670. 10.1037/dev0000906. 32077732 PMC7041962

[sjop70082-bib-0024] Eisenberg, N. , A. Cumberland , and T. L. Spinrad . 1998. “Parental Socialization of Emotion.” Psychological Inquiry 9, no. 4: 241–273. 16865170 10.1207/s15327965pli0904_1 PMC1513625

[sjop70082-bib-0025] Endendijk, J. J. , M. G. Groeneveld , M. J. Bakermans‐Kranenburg , and J. Mesman . 2016. “Gender‐Differentiated Parenting Revisited: Meta‐Analysis Reveals Very Few Differences in Parental Control of Boys and Girls.” PLoS One 11, no. 7: e0159193. 10.1371/journal.pone.0159193. 27416099 PMC4945059

[sjop70082-bib-0026] Erel, O. , and B. Burman . 1995. “Interrelatedness of Marital Relations and Parent‐Child Relations: A Meta‐Analytic Review.” Psychological Bulletin 118, no. 1: 108–132. 10.1037/0033-2909.118.1.108. 7644602

[sjop70082-bib-0027] Fabes, R. A. , S. A. Leonard , K. Kupanoff , and C. L. Martin . 2001. “Parental Coping With Children's Negative Emotions: Relations With Children's Emotional and Social Responding.” Child Development 72, no. 3: 907–920. 11405590 10.1111/1467-8624.00323

[sjop70082-bib-0028] Freisthler, B. , and P. J. Gruenewald . 2013. “Where the Individual Meets the Ecological: A Study of Parent Drinking Patterns, Alcohol Outlets, and Child Physical Abuse.” Alcoholism: Clinical and Experimental Research 37, no. 6: 993–1000. 10.1111/acer.12059. 23316780 PMC3628976

[sjop70082-bib-0029] Frogley, W. J. , G. L. King , and E. M. Westrupp . 2023. “Profiles of Parent Emotion Socialization: Longitudinal Associations With Child Emotional Outcomes.” Mental Health & Prevention 30: 200274. 10.1016/j.mhp.2023.200274.

[sjop70082-bib-0030] Fülöp, F. , B. Bőthe , É. Gál , J. Y. A. Cachia , Z. Demetrovics , and G. Orosz . 2022. “A Two‐Study Validation of a Single‐Item Measure of Relationship Satisfaction: RAS‐1.” Current Psychology 41, no. 4: 2109–2121. 10.1007/s12144-020-00727-y.

[sjop70082-bib-0100] Garside, R. B. , and B. Klimes‐Dougan . 2002. “Socialization of Discrete Negative Emotions: Gender Differences and Links with Psychological Distress.” Sex Roles 47, no. 3‐4: 115–128. 10.1023/A:1021090904785.

[sjop70082-bib-0031] Gaylord‐Harden, N. , J. Cunningham , G. Holmbeck , and K. Grant . 2010. “Suppressor Effects in Coping Research With African American Adolescents From Low‐Income Communities.” Journal of Consulting and Clinical Psychology 78: 843–855. 10.1037/a0020063. 20873890

[sjop70082-bib-0032] Godleski, S. A. , R. D. Eiden , S. Shisler , and J. A. Livingston . 2020. “Parent Socialization of Emotion in a High‐Risk Sample.” Developmental Psychology 56, no. 3: 489–502. 10.1037/dev0000793. 32077719 PMC7041846

[sjop70082-bib-0033] Goetz, L. H. , and N. J. Schork . 2018. “Personalized Medicine: Motivation, Challenges, and Progress.” Fertility and Sterility 109, no. 6: 952–963. 10.1016/j.fertnstert.2018.05.006. 29935653 PMC6366451

[sjop70082-bib-0034] Gottman, J. M. , L. F. Katz , and C. Hooven . 1996. “Parental Meta‐Emotion Philosophy and the Emotional Life of Families: Theoretical Models and Preliminary Data.” Journal of Family Psychology 10, no. 3: 243–268.

[sjop70082-bib-0035] Hajal, N. J. , and B. Paley . 2020. “Parental Emotion and Emotion Regulation: A Critical Target of Study for Research and Intervention to Promote Child Emotion Socialization.” Developmental Psychology 56, no. 3: 403–417. 10.1037/dev0000864. 32077713

[sjop70082-bib-0036] Hendrick, S. S. 1988. “A Generic Measure of Relationship Satisfaction.” Journal of Marriage and the Family 50, no. 1: 93–98. 10.2307/352430.

[sjop70082-bib-0037] Hesbacher, P. T. , K. Rickels , R. J. Morris , H. Newman , and H. Rosenfeld . 1980. “Psychiatric Illness in Family Practice.” Journal of Clinical Psychiatry 41, no. 1: 6–10. 7351399

[sjop70082-bib-0038] Hofmann, S. G. , and S. N. Doan . 2018. “Socialization of Emotions.” In The Social Foundations of Emotion: Developmental, Cultural, and Clinical Dimensions, 81–101. American Psychological Association. 10.1037/0000098-006.

[sjop70082-bib-0096] Huffhines, L. , L. G. Bailes , J. L. Coe , E. M. Leerkes , and S. H. Parade . 2024. “Mothers’ Recollections of Parenting by Their Own Mothers and Fathers and Maternal and Infant Outcomes.” Family Relations 73, no. 3: 1949–1967. 10.1111/fare.12988. 39545098 PMC11562778

[sjop70082-bib-0099] Hughes, E. K. , and E. Gullone . 2010. “Parent Emotion Socialisation Practices and Their Associations with Personality and Emotion Regulation.” Personality and Individual Differences 49, no. 7: 694–699. 10.1016/j.paid.2010.05.042.

[sjop70082-bib-0039] Johnson, A. M. , D. J. Hawes , N. Eisenberg , J. Kohlhoff , and J. Dudeney . 2017. “Emotion Socialization and Child Conduct Problems: A Comprehensive Review and Meta‐Analysis.” Clinical Psychology Review 54: 65–80. 10.1016/j.cpr.2017.04.001. 28414957

[sjop70082-bib-0040] Johnston, R. , K. Jones , and D. Manley . 2018. “Confounding and Collinearity in Regression Analysis: A Cautionary Tale and an Alternative Procedure, Illustrated by Studies of British Voting Behaviour.” Quality & Quantity 52, no. 4: 1957–1976. 10.1007/s11135-017-0584-6. 29937587 PMC5993839

[sjop70082-bib-0041] Keller, P. S. , E. Mark Cummings , and P. T. Davies . 2005. “The Role of Marital Discord and Parenting in Relations Between Parental Problem Drinking and Child Adjustment.” Journal of Child Psychology and Psychiatry 46, no. 9: 943–951. 10.1111/j.1469-7610.2004.00399.x. 16108997

[sjop70082-bib-0042] Kendler, K. S. , and J. H. Baker . 2007. “Genetic Influences on Measures of the Environment: A Systematic Review.” Psychological Medicine 37: 615. 10.1017/S0033291706009524. 17176502

[sjop70082-bib-0043] Kerr, D. C. R. , and D. M. Capaldi . 2019. “Intergenerational Transmission of Parenting.” In Handbook of Parenting, 3rd ed. Routledge.

[sjop70082-bib-0044] Kiff, C. J. , L. J. Lengua , and M. Zalewski . 2011. “Nature and Nurturing: Parenting in the Context of Child Temperament.” Clinical Child and Family Psychology Review 14, no. 3: 251–301. 10.1007/s10567-011-0093-4. 21461681 PMC3163750

[sjop70082-bib-0045] Kim, J. H. , and S. Wang . 2023. “Birth Order Effects and Parenting Behaviors.” China Economic Review 79: 101950. 10.1016/j.chieco.2023.101950.

[sjop70082-bib-0046] King, G. L. , J. A. Macdonald , C. J. Greenwood , et al. 2023. “Profiles of Parents' Emotion Socialization Within a Multinational Sample of Parents.” Frontiers in Psychology 14: 1161418. 10.3389/fpsyg.2023.1161418. 37637929 PMC10447894

[sjop70082-bib-0047] Klahr, A. M. , and S. A. Burt . 2014. “Elucidating the Etiology of Individual Differences in Parenting: A Meta‐Analysis of Behavioral Genetic Research.” Psychological Bulletin 140, no. 2: 544–586. 10.1037/a0034205. 24016230

[sjop70082-bib-0048] Kretschmer, T. 2023. “Parenting Is Genetically Influenced: What Does That Mean for Research Into Child and Adolescent Social Development?” Social Development 32, no. 1: 3–16. 10.1111/sode.12633.

[sjop70082-bib-0097] Leerkes, E. M. , L. G. Bailes , and M. E. Augustine . 2020. “The Intergenerational Transmission of Emotion Socialization.” Developmental Psychology 56, no. 3: 0012–1649. 10.1037/dev0000753. 32077712

[sjop70082-bib-0049] Leerkes, E. M. , and K. J. Siepak . 2006. “Attachment Linked Predictors of Women's Emotional and Cognitive Responses to Infant Distress.” Attachment & Human Development 8, no. 1: 11–32. 10.1080/14616730600594450. 16581621

[sjop70082-bib-0050] Louie, A. D. , L. D. Cromer , and J. O. Berry . 2017. “Assessing Parenting Stress: Review of the Use and Interpretation of the Parental Stress Scale.” Family Journal 25, no. 4: 359–367. 10.1177/1066480717731347.

[sjop70082-bib-0051] Lovejoy, M. , P. A. Graczyk , E. O'Hare , and G. Neuman . 2000. “Maternal Depression and Parenting Behavior: A Meta‐Analytic Review.” Clinical Psychology Review 20, no. 5: 561–592. 10.1016/S0272-7358(98)00100-7. 10860167

[sjop70082-bib-0052] Lugo‐Candelas, C. I. , E. A. Harvey , and R. P. Breaux . 2015. “Emotion Socialization Practices in Latina and European‐American Mothers of Preschoolers With Behavior Problems.” Journal of Family Studies 21, no. 2: 144–162. 10.1080/13229400.2015.1020982. 27042157 PMC4813662

[sjop70082-bib-0053] Lunkenheimer, E. S. , A. M. Shields , and K. S. Cortina . 2007. “Parental Emotion Coaching and Dismissing in Family Interaction.” Social Development 16, no. 2: 232–248. 10.1111/j.1467-9507.2007.00382.x.

[sjop70082-bib-0055] McGuire, S. , N. L. Segal , and S. Hershberger . 2012. “Parenting as Phenotype: A Behavioral Genetic Approach to Understanding Parenting.” Parenting 12: 192–201. 10.1080/15295192.2012.683357. 23144594 PMC3491356

[sjop70082-bib-0056] McKee, L. G. , K. DiMarzio , J. Parent , C. Dale , J. Acosta , and J. O'Leary . 2022. “Profiles of Emotion Socialization Across Development and Longitudinal Associations With Youth Psychopathology.” Research on Child and Adolescent Psychopathology 50, no. 2: 193–210. 10.1007/s10802-021-00829-6. 34081230 PMC8639825

[sjop70082-bib-0057] Milan, S. , C. Carlone , and A. L. B. T. Dáu . 2021. “Emotion Socialization in Mothers Who Experienced Maltreatment: Mentalizing the Past May Help the Present.” Journal of Family Psychology 35, no. 6: 851–856. 10.1037/fam0000839. 33705171

[sjop70082-bib-0058] Mönkediek, B. , W. Schulz , H. Eichhorn , and M. Diewald . 2020. “Is There Something Special About Twin Families? A Comparison of Parenting Styles in Twin and Non‐Twin Families.” Social Science Research 90: 102441. 10.1016/j.ssresearch.2020.102441. 32825925

[sjop70082-bib-0059] Morin, A. J. S. , J.‐S. Boudrias , H. W. Marsh , et al. 2017. “Complementary Variable‐ and Person‐Centered Approaches to the Dimensionality of Psychometric Constructs: Application to Psychological Wellbeing at Work.” Journal of Business and Psychology 32, no. 4: 395–419. 10.1007/s10869-016-9448-7.

[sjop70082-bib-0060] Morris, A. S. , L. Cui , J. E. Jespersen , M. M. Criss , and K. T. Cosgrove . 2022. “Parenting and Children's Social and Emotional Development: Emotion Socialization Across Childhood and Adolescence.” In The Cambridge Handbook of Parenting, edited by A. S. Morris and J. Mendez Smith , 1st ed., 71–94. Cambridge University Press. 10.1017/9781108891400.006.

[sjop70082-bib-0061] Morris, A. S. , J. S. Silk , L. Steinberg , S. S. Myers , and L. R. Robinson . 2007. “The Role of the Family Context in the Development of Emotion Regulation.” Social Development 16, no. 2: 361–388. 10.1111/j.1467-9507.2007.00389.x. 19756175 PMC2743505

[sjop70082-bib-0062] Nakagawa, S. , and H. Schielzeth . 2013. “A General and Simple Method for Obtaining R2 From Generalized Linear Mixed‐Effects Models.” Methods in Ecology and Evolution 4, no. 2: 133–142. 10.1111/j.2041-210x.2012.00261.x.

[sjop70082-bib-0063] Nelson, J. A. , A. B. Aguas , and J. M. Katz . 2023. ““Work Through It on Your Own”: Parent‐Centered Emotion Socialization Beliefs and Parenting Stress.” Family Process 63, no. 3: 1521–1530. 10.1111/famp.12939. 37705166

[sjop70082-bib-0064] Nelson, J. A. , M. O'Brien , A. N. Blankson , S. D. Calkins , and S. P. Keane . 2009. “Family Stress and Parental Responses to Children's Negative Emotions: Tests of the Spillover, Crossover, and Compensatory Hypotheses.” Journal of Family Psychology 23, no. 5: 671–679. 10.1037/a0015977. 19803603 PMC2855124

[sjop70082-bib-0065] Patterson, G. R. 2016. “Coercion Theory: The Study of Change.” In The Oxford Handbook of Coercive Relationship Dynamics, 7–22. Oxford University Press.

[sjop70082-bib-0066] Paulhus, D. L. 1984. “Two‐Component Models of Socially Desirable Responding.” Journal of Personality and Social Psychology 46, no. 3: 598–609.

[sjop70082-bib-0067] Plomin, R. 2023. “Nonshared Environment: Real but Random.” JCPP Advances 4: e12229. 10.1002/jcv2.12229. PMC11472802 39411468

[sjop70082-bib-0068] Price, J. 2008. “Parent‐Child Quality Time: Does Birth Order Matter?” Journal of Human Resources 43, no. 1: 240–265. 10.1353/jhr.2008.0023.

[sjop70082-bib-0069] Prinzie, P. , G. J. J. M. Stams , M. Deković , A. H. A. Reijntjes , and J. Belsky . 2009. “The Relations Between Parents' Big Five Personality Factors and Parenting: A Meta‐Analytic Review.” Journal of Personality and Social Psychology 97, no. 2: 351–362. 10.1037/a0015823. 19634980

[sjop70082-bib-0070] R Core Team . 2025. R: A Language and Environment for Statistical Computing [Computer software]. R Foundation for Statistical Computing. https://www.R‐project.org.

[sjop70082-bib-0071] Raval, V. V. , and B. L. Walker . 2019. “Unpacking ‘Culture’: Caregiver Socialization of Emotion and Child Functioning in Diverse Families.” Developmental Review 51: 146–174. 10.1016/j.dr.2018.11.001.

[sjop70082-bib-0072] Root, A. K. , and S. A. Denham . 2010. “The Role of Gender in the Socialization of Emotion: Key Concepts and Critical Issues.” New Directions for Child and Adolescent Development 2010, no. 128: 1–9. 10.1002/cd.265. 20552661

[sjop70082-bib-0101] Rossi, A. S. 1980. “Life‐Span Theories and Women's Lives.” Journal of Women in Culture and Society 6, no. 1: 4–32. 10.1086/493773.

[sjop70082-bib-0073] Rothenberg, W. A. 2019. “Intergenerational Continuity in Parenting: A Review and Theoretical Integration.” Marriage & Family Review 55, no. 8: 8. 10.1080/01494929.2019.1589618.

[sjop70082-bib-0074] Scott, S. A. , and J. Hakim‐Larson . 2021. “Temperament, Emotion Regulation, and Emotion‐Related Parenting: Maternal Emotion Socialization During Early Childhood.” Journal of Child and Family Studies 30, no. 10: 2353–2366. 10.1007/s10826-021-02016-z.

[sjop70082-bib-0075] Shaffer, A. , C. Suveg , K. Thomassin , and L. L. Bradbury . 2012. “Emotion Socialization in the Context of Family Risks: Links to Child Emotion Regulation.” Journal of Child and Family Studies 21, no. 6: 917–924. 10.1007/s10826-011-9551-3.

[sjop70082-bib-0076] Shewark, E. A. , A. M. Ramos , C. Liu , et al. 2021. “The Role of Child Negative Emotionality in Parenting and Child Adjustment: Gene‐Environment Interplay.” Journal of Child Psychology and Psychiatry, and Allied Disciplines 62, no. 12: 1453–1461. 10.1111/jcpp.13420. 33821495 PMC8492791

[sjop70082-bib-0077] Siddiqui, K. 2013. Heuristics for Sample Size Determination in Multivariate Statistical Techniques.

[sjop70082-bib-0078] Sosa‐Hernandez, L. , L. Sack , J. A. Seddon , K. Bailey , and K. Thomassin . 2020. “Mother and Father Repertoires of Emotion Socialization Practices in Middle Childhood.” Journal of Applied Developmental Psychology 69: 101159. 10.1016/j.appdev.2020.101159.

[sjop70082-bib-0079] Soto, C. J. , and O. P. John . 2017. “Short and Extra‐Short Forms of the Big Five Inventory–2: The BFI‐2‐S and BFI‐2‐XS.” Journal of Research in Personality 68: 69–81. 10.1016/j.jrp.2017.02.004.

[sjop70082-bib-0080] Statistics Norway . 2024. “SSB.” Accessed June 3, 2025. https://www.ssb.no/befolkning/likestilling/statistikk/indikatorer‐for‐kjonnslikestilling‐i‐kommunene/artikler/hvor‐mange‐tar‐ut‐pappaperm.

[sjop70082-bib-0081] Sulloway, F. J. 2007. “Birth Order and Intelligence.” Science 316, no. 5832: 1711–1712. 10.1126/science.1144749. 17588922

[sjop70082-bib-0098] Tadjine, S. , and L. Swords . 2024. ““I Just Wouldn't Like Him to go Through What I Went Through as a Kid”: A Qualitative Study on the Mitigating Effects of Positive Childhood Experiences in Mothers with a History of Adverse Childhood Experiences in an Irish Population.” Community Mental Health Journal 61: 492–501. 10.1007/s10597-024-01353-9. 39277558 PMC11868248

[sjop70082-bib-0082] Taraban, L. , and D. S. Shaw . 2018. “Parenting in Context: Revisiting Belsky's Classic Process of Parenting Model in Early Childhood.” Developmental Review 48: 55–81. 10.1016/j.dr.2018.03.006.

[sjop70082-bib-0083] Thalmayer, A. G. , G. Saucier , and A. Eigenhuis . 2011. “Comparative Validity of Brief to Medium‐Length Big Five and Big Six Personality Questionnaires.” Psychological Assessment 23, no. 4: 995–1009. 10.1037/a0024165. 21859221

[sjop70082-bib-0084] Thompson, S. F. , M. Zalewski , C. J. Kiff , L. Moran , R. Cortes , and L. J. Lengua . 2020. “An Empirical Test of the Model of Socialization of Emotion: Maternal and Child Contributors to Preschoolers' Emotion Knowledge and Adjustment.” Developmental Psychology 56, no. 3: 418–430. 10.1037/dev0000860. 32077714 PMC7041859

[sjop70082-bib-0085] Trifan, T. A. , H. Stattin , and L. Tilton‐Weaver . 2014. “Have Authoritarian Parenting Practices and Roles Changed in the Last 50 Years?” Journal of Marriage and Family 76, no. 4: 744–761. 10.1111/jomf.12124.

[sjop70082-bib-0086] Wong, M. S. , N. L. McElwain , and A. G. Halberstadt . 2009. “Parent, Family, and Child Characteristics: Associations With Mother‐ and Father‐Reported Emotion Socialization Practices.” Journal of Family Psychology 23, no. 4: 452. 10.1037/a0015552. 19685981

[sjop70082-bib-0087] World Economic Forum . n.d. “Global Gender Gap Report 2023.” World Economic Forum. https://www.weforum.org/publications/global‐gender‐gap‐report‐2023/.

[sjop70082-bib-0088] World Health Organization . 2025. “Mental Health.” Accessed May 27, 2025. https://www.who.int/health‐topics/mental‐health.

[sjop70082-bib-0089] Yaffe, Y. 2023. “Systematic Review of the Differences Between Mothers and Fathers in Parenting Styles and Practices.” Current Psychology 42, no. 19: 16011–16024. 10.1007/s12144-020-01014-6.

[sjop70082-bib-0090] Zahn‐Waxler, C. 2010. “Socialization of Emotion: Who Influences Whom and How?” New Directions for Child and Adolescent Development 2010, no. 128: 101–109. 10.1002/cd.271. 20552660

[sjop70082-bib-0091] Zeman, J. , C. Perry‐Parrish , and M. Cassano . 2010. “Parent‐Child Discussions of Anger and Sadness: The Importance of Parent and Child Gender During Middle Childhood.” New Directions for Child and Adolescent Development 2010, no. 128: 65–83. 10.1002/cd.269. 20552662

