# Supplementary material for: Determinants of Parental Emotion Socialization Behaviors: Insights From a Large‐Scale Population‐Based Family Study
Source: Scand J Psychol. 2026 Feb 18;67(4):953–71. doi: 10.1111/sjop.70082 (PMC13352549; doi:10.1111/sjop.70082)
Supplement: Supplementary file 1 — Data S1: sjop70082‐sup‐0001‐DataS1.docx. [file SJOP-67-953-s001.docx]

**Supplementary Material**

**Research question**

In addition to the main research questions—examining the relative contribution of the three predictor domains and identifying the most influential predictors of emotion socialization behaviors (ESBs)—we conducted supplementary analyses to estimate the models separately for mothers and fathers. These analyses aimed to provide insight into whether the three predictor groups showed similar patterns of association with supportive and non-supportive ESBs across parent groups. They were not intended to formally test gender differences, but to increase transparency and offer a more complete overview of the data.

**Statistical analyses**

The supplementary analyses followed the same analytic strategy as described in the main manuscript and were conducted using R (version 4.4.3; R Core Team, 2025). Supportive and non-supportive ESBs were analyzed separately for mothers and fathers, using a four-step hierarchical regression procedure to test three predictor domains: the parent, child, and family contexts. As in the primary models, kinship was included as a random intercept to account for the relatedness among family members. Consistent with the main analyses, we report marginal R^2^ as an estimate of variance explained by fixed effects, and the intraclass correlation (ICC) as a null model with intraclass correlation coefficient (ICC) to quantify the variance attributable to family-level clustering.

**Results of separate hierarchical regression analyses for mothers and fathers.**

Results from the separate hierarchical regression analyses for mothers and fathers are presented along with descriptive characteristics of each sub-sample. Table S2 presents findings for supportive ESBs, and Table S3 reports results for non-supportive ESBs.

Overall, the relative importance of the three predictor groups —parent, context, and child factors —is essentially the same as observed in the full sample. Parent factors account for most of the variance for both mothers and fathers, with recollected ESBsfrom their upbringing, and the personality traits agreeableness and openness emerging as the strongest predictors. Higher scores on agreeableness and openness are consistently associated with more supportive and fewer non-supportive ESBs.

Although these analyses were not intended to formally test gender differences, the subgroup models show some variation. The full models explained more variance in fathers than in mothers, for both supportive and non-supportive ESBs. There was also some variation in which predictors reached significance within each group. For example, regarding parent factors, symptoms of anxiety and depression predict less use of non-supportive strategies only for fathers, while these symptoms predicted increased use of supportive ESBs in both groups. Among family context factors, birth country was a significant predictor only in the models for mothers, and for both supportive and non-supportive strategies. Education showed the same pattern in both groups, with higher levels predicting lower levels of non-supportive ESBs; income was only predictive of mothers’ use of non-supportive ESBs, with higher income predicting more use of non-supportive strategies. Regarding child factors, higher child age and birth order were associated with lower use of supportive ESBs among both mothers and fathers. For non-supportive ESBs, child age was the only significant predictor, and only for mothers.

Across all models, the intraclass correlations (ICCs) ranged from .006 to .108, indicating modest family-level clustering, with a general pattern of higher estimates in the models for mothers compared to fathers, particularly for non-supportive ESBs.

**Table S1**

*Descriptive Statistics for Sample Characteristics and Study Instruments Separately for Mothers and Fathers*

| **Characteristics** | **Mothers** | | **Fathers** | |
| --- | --- | --- | --- | --- |
|  | ***n* (%) or M (SD)** | **Range** | ***n* (%) or M (SD)** | **Range** |
| Sample size | 2460 (58) |  | 1747 (42) |  |
| Longest completed education |  |  |  |  |
| Elementary or vocational college | 564 (23) |  | 655 (38) |  |
| College or university < 4 years | 901 (37) |  | 490 (28) |  |
| College or university > 4 years | 995 (40) |  | 602 (34) |  |
| Personal income (NOK) |  |  |  |  |
| Low income (< 300 000) | 205 (8) |  | 21 (1) |  |
| 300 000 – 500 000 | 653 (27) |  | 160 (9) |  |
| 500 000 – 700 000 | 1062 (43) |  | 610 (35) |  |
| 700 000 – 100 000 | 390 (16) |  | 575 (33) |  |
| Above 1 000 000 | 150 (6) |  | 381 (22) |  |
| Number of children in the household |  |  |  |  |
| 0 ^a^ | 1 (<1) |  | 4 (<1) |  |
| 1 | 184 (8) |  | 146 (8) |  |
| 2 | 1331 (54) |  | 930 (53) |  |
| 3 | 697 (28) |  | 499 (29) |  |
| > 4 | 247 (10) |  | 172 (10) |  |
| Birth country |  |  |  |  |
| Norway | 2262 (92) |  | 1587 (91) |  |
| Other | 198 (8) |  | 160 (9) |  |
| Child gender |  |  |  |  |
| Female | 1230 (50) |  | 900 (52) |  |
| Male | 1230 (50) |  | 847 (48) |  |
| Child birth-order |  |  |  |  |
| First | 1061 (43) |  | 771 (44) |  |
| Second | 915 (38) |  | 633 (36) |  |
| Third | 398 (16) |  | 276 (16) |  |
| Fourth | 71 (<1) |  | 55 (3) |  |
| >Fifth | 15 (<1) |  | 12 (<1) |  |
| Parent age (years) | 39.1 (4.94) | 25–58 | 41.3 (5.29) | 27–61 |
| Child age (years) | 7.4 (2.23) | 3–13 | 7.4 (2.23) | 3–13 |
| Supportive ESB | 5.8 (0.91) | 1–7 | 5.2 (1.00) | 1–7 |
| Non-supportive ESB | 2.0 (0.71) | 1–6 | 2.5 (1.10) | 1–7 |
| Parental stress | 2.1 (0.57) | 1–4.43 | 2.0 (0.52) | 1–3.86 |
| Anxiety & depression | 1.5 (0.52) | 1–4 | 1.4 (0.46) | 1–4 |
| Alcohol use | 3.3 (1.89) | 0–10 | 4.4 (2.06) | 0–12 |
| Extraversion | 3.8 (0.77) | 1.33–5 | 3.6 (0.77) | 1–5 |
| Agreeableness | 4.2 (0.55) | 1.33–5 | 3.9 (0.60) | 1.67–5 |
| Conscientiousness | 4.1 (0.76) | 1.5–5 | 3.8 (0.78) | 1.33–5 |
| Neuroticism | 2.6 (0.83) | 1–5 | 2.3 (0.76) | 1–5 |
| Openness | 3.7 (0.77) | 1–5 | 3.7 (0.72) | 1–5 |
| Recollected supportive ESB | 3.5 (1.57) | 1–7 | 3.7 (1.40) | 1–7 |
| Recollected non-sup. ESB | 3.0 (1.34) | 1–7 | 3.0 (1.07) | 1–7 |
| Relationship satisfaction | 4.3 (0.86) | 1–5 | 4.3 (0.89) | 1–5 |
| Negative emotionality | 3.3 (0.89) | 1–5 | 3.3 (0.84) | 1–5 |

*Note. Total N =* 4207. All continuous variables have complete data. M = mean; SD = standard deviation; Recollected non-sup. ESBs= Recollected non-supportive ESBs.

^a^ Reflects that five parents in total reported on a child who does not live in their household

**Table S2**

*Hierarchical Regression Results for Supportive ESBs separately for Mothers and Fathers*

| **Variable** | **Mothers (***n* = 2460) | | | | |  | **Fathers (***n* = 1746) | | | | |
| --- | --- | --- | --- | --- | --- | --- | --- | --- | --- | --- | --- |
|  | ***β*** | **SE *β*** | **R²_m_** | **𝚫 R²_m_** | **ICC** |  | ***β*** | **SE *β*** | **R²_m_** | **𝚫 R²_m_** | **ICC** |
| **Step 1 *Null model^a^*** |  |  |  |  | .068 |  |  |  |  |  | .006 |
| Constant | .23 | .02 |  |  |  |  | -.31 | .02 |  |  |  |
| **Step 2 *Parent factors*** |  |  | .173 | .173** | .079 |  |  |  | .285 | .285** | .038 |
| Constant | .16 | .02 |  |  |  |  | -.24 | .03 |  |  |  |
| Parent age | -.04 | .02 |  |  |  |  | -.03 | .02 |  |  |  |
| Parental stress | -.07** | .02 |  |  |  |  | -.08** | .03 |  |  |  |
| Anx. & depr. | .10** | .02 |  |  |  |  | .11** | .03 |  |  |  |
| Alcohol use | -.08** | .02 |  |  |  |  | -.09** | .02 |  |  |  |
| Extraversion | .02 | .02 |  |  |  |  | .02 | .02 |  |  |  |
| Agreeableness | .19** | .02 |  |  |  |  | .21** | .02 |  |  |  |
| Conscientiousness | .02 | .02 |  |  |  |  | .01 | .02 |  |  |  |
| Neuroticism | .00 | .02 |  |  |  |  | -.06 | .03 |  |  |  |
| Openness | .10** | .02 |  |  |  |  | .12** | .02 |  |  |  |
| Rec. supportive ESB | .28** | .02 |  |  |  |  | .45** | .02 |  |  |  |
| Rec. non-sup. ESB | .22** | .02 |  |  |  |  | .18** | .03 |  |  |  |
| **Step 3 *Family context*** |  |  | .178 | .005* | .074 |  |  |  | .287 | .002 | . 038 |
| Constant | .16 | .02 |  |  |  |  | -.24 | .03 |  |  |  |
| Parent age | -.04 | .02 |  |  |  |  | -.03 | .02 |  |  |  |
| Parental stress | -.06** | .02 |  |  |  |  | -.07** | .03 |  |  |  |
| Anx. & depr. | .09** | .02 |  |  |  |  | .11** | .03 |  |  |  |
| Alcohol use | -.08** | .02 |  |  |  |  | -.10** | .02 |  |  |  |
| Extraversion | .03 | .02 |  |  |  |  | .02 | .02 |  |  |  |
| Agreeableness | .19** | .02 |  |  |  |  | .20** | .02 |  |  |  |
| Conscientiousness | .02 | .02 |  |  |  |  | .01 | .02 |  |  |  |
| Neuroticism | .00 | .02 |  |  |  |  | -.06 | .03 |  |  |  |
| Openness | .09** | .02 |  |  |  |  | .12** | .02 |  |  |  |
| Rec. supportive ESB | .28** | .02 |  |  |  |  | .45** | .02 |  |  |  |
| Rec. non-sup. ESB | .22** | .02 |  |  |  |  | .18** | .03 |  |  |  |
| Education | .02 | .02 |  |  |  |  | -.02 | .02 |  |  |  |
| Income | -.05 | .03 |  |  |  |  | .00 | .02 |  |  |  |
| Rel. satisfaction | .02 | .02 |  |  |  |  | .00 | .02 |  |  |  |
| Number of children | -.04* | .02 |  |  |  |  | -.04 | .02 |  |  |  |
| Birth country | -.17* | .06 |  |  |  |  | -.03 | .07 |  |  |  |
| **Step 4 *Child factors*** |  |  | .184 | .006** | .072 |  |  |  | .298 | .011** | .040 |
| Constant | .14 | .05 |  |  |  |  | -.18 | .07 |  |  |  |
| Parent age | .02 | .02 |  |  |  |  | .04 | .03 |  |  |  |
| Parental stress | -.07** | .02 |  |  |  |  | -.10** | .03 |  |  |  |
| Anx. & depr. | .09** | .02 |  |  |  |  | .11** | .03 |  |  |  |
| Alcohol use | -.08** | .02 |  |  |  |  | -.10** | .02 |  |  |  |
| Extraversion | .03 | .02 |  |  |  |  | .03 | .02 |  |  |  |
| Agreeableness | .18** | .02 |  |  |  |  | .20** | .02 |  |  |  |
| Conscientiousness | .02 | .02 |  |  |  |  | .01 | .02 |  |  |  |
| Neuroticism | -.01 | .02 |  |  |  |  | -.07* | .03 |  |  |  |
| Openness | .09** | .02 |  |  |  |  | .12** | .02 |  |  |  |
| Rec. supportive ESB | .28** | .02 |  |  |  |  | .45** | .02 |  |  |  |
| Rec. non-sup. ESB | .23** | .02 |  |  |  |  | .18** | .03 |  |  |  |
| Education | .01 | .02 |  |  |  |  | -.03 | .02 |  |  |  |
| Income | -.05 | .03 |  |  |  |  | .00 | .02 |  |  |  |
| Rel. satisfaction | .02 | .02 |  |  |  |  | .00 | .02 |  |  |  |
| Number of children | -.01 | .02 |  |  |  |  | -.01 | .02 |  |  |  |
| Birth country | -.18* | .06 |  |  |  |  | -.05 | .07 |  |  |  |
| Child Age | -.05* | .02 |  |  |  |  | -.07** | .02 |  |  |  |
| Child Gender | .02 | .03 |  |  |  |  | -.05 | .04 |  |  |  |
| Birth order | -.07** | .02 |  |  |  |  | -.08** | .03 |  |  |  |
| Negative emotionality | .00 | .02 |  |  |  |  | -.03 | .02 |  |  |  |

*Note*. Rel. satisfaction. = Relationship satisfaction, Anx. & Depr. = Anxiety & Depression, Rec. supportive ESBs= Recollected supportive ESB, Rec. non-s. ESBs= Recollected non-supportive ESB, **p* < .01, ***p* < .001.

^a^ = including only a random intercept accounting for family-level clustering.

**Table S3**

*Hierarchical Regression Results for Non-supportive ESBs separately for Mothers and Fathers*

| **Variable** | **Mothers (***n* = 2460) | | | | |  | **Fathers (***n* = 1746) | | | | |
| --- | --- | --- | --- | --- | --- | --- | --- | --- | --- | --- | --- |
|  | ***β*** | **SE *β*** | **R²_m_** | **𝚫 R²_m_** | **ICC** |  | ***β*** | **SE *β*** | **R²_m_** | **𝚫 R²_m_** | **ICC** |
| **Step 1 *Null model^a^*** |  |  |  |  | .102 |  |  |  |  |  | .077 |
| Constant | -.26 | .02 |  |  |  |  | .36 | .02 |  |  |  |
| **Step 2 *Parent factors*** |  |  | .170 | .170** | .108 |  |  |  | .258 | .258** | .034 |
| Constant | -.20 | .02 |  |  |  |  | .27 | .02 |  |  |  |
| Parent age | .07** | .02 |  |  |  |  | .05* | .02 |  |  |  |
| Parental stress | .08** | .02 |  |  |  |  | .11** | .02 |  |  |  |
| Anx. & depr. | -.01 | .02 |  |  |  |  | -.08** | .03 |  |  |  |
| Alcohol use | .05 | .02 |  |  |  |  | .07** | .02 |  |  |  |
| Extraversion | .05 | .02 |  |  |  |  | .01 | .02 |  |  |  |
| Agreeableness | -.16** | .02 |  |  |  |  | -.17** | .02 |  |  |  |
| Conscientiousness | .03 | .02 |  |  |  |  | .02 | .02 |  |  |  |
| Neuroticism | .00 | .02 |  |  |  |  | .00 | .03 |  |  |  |
| Openness | -.09** | .02 |  |  |  |  | -.09** | .02 |  |  |  |
| Rec. supportive ESB | .28** | .02 |  |  |  |  | .23** | .02 |  |  |  |
| Rec. non-sup. ESB | .34** | .02 |  |  |  |  | .54** | .03 |  |  |  |
| **Step 3 *Family context*** |  |  | .185 | .015** | . 088 |  |  |  | .266 | .009** | 027 |
| Constant | -.19 | .02 |  |  |  |  | .23 | .03 |  |  |  |
| Parent age | .08** | .02 |  |  |  |  | .06** | .02 |  |  |  |
| Parental stress | .08** | .02 |  |  |  |  | .10** | .02 |  |  |  |
| Anx. & depr. | -.01 | .02 |  |  |  |  | -.07* | .03 |  |  |  |
| Alcohol use | .06** | .02 |  |  |  |  | .07** | .02 |  |  |  |
| Extraversion | .04 | .02 |  |  |  |  | .00 | .02 |  |  |  |
| Agreeableness | -.17** | .02 |  |  |  |  | -.17** | .02 |  |  |  |
| Conscientiousness | .03 | .02 |  |  |  |  | .02 | .02 |  |  |  |
| Neuroticism | .01 | .02 |  |  |  |  | .00 | .03 |  |  |  |
| Openness | -.09** | .02 |  |  |  |  | -.09** | .02 |  |  |  |
| Rec. supportive ESB | .27** | .02 |  |  |  |  | .23** | .02 |  |  |  |
| Rec. non-sup. ESB | .32** | .02 |  |  |  |  | .53** | .03 |  |  |  |
| Education | -.09** | .02 |  |  |  |  | -.08** | .02 |  |  |  |
| Income | .06 | .03 |  |  |  |  | .03 | .02 |  |  |  |
| Rel. satisfaction | .00 | .02 |  |  |  |  | .00 | .02 |  |  |  |
| Number of children | .05* | .02 |  |  |  |  | .05 | .02 |  |  |  |
| Birth country | .27** | .06 |  |  |  |  | .12 | .07 |  |  |  |
| **Step 4 *Child factors*** |  |  | .190 | .004* | .089 |  |  |  | .283 | .017** | .021 |
| Constant | -.18 | .05 |  |  |  |  | .21 | .07 |  |  |  |
| Parent age | .04 | .02 |  |  |  |  | -.01 | .03 |  |  |  |
| Parental stress | .08** | .02 |  |  |  |  | .12** | .03 |  |  |  |
| Anx. & depr. | -.01 | .02 |  |  |  |  | -.07* | .03 |  |  |  |
| Alcohol use | .05** | .02 |  |  |  |  | .08** | .02 |  |  |  |
| Extraversion | .04 | .02 |  |  |  |  | .00 | .02 |  |  |  |
| Agreeableness | -.16** | .02 |  |  |  |  | -.16** | .02 |  |  |  |
| Conscientiousness | .03 | .02 |  |  |  |  | .02 | .02 |  |  |  |
| Neuroticism | .01 | .02 |  |  |  |  | .00 | .03 |  |  |  |
| Openness | -.09** | .02 |  |  |  |  | -.08** | .02 |  |  |  |
| Rec. supportive ESB | .27** | .02 |  |  |  |  | .23** | .02 |  |  |  |
| Rec. non-sup. ESB | .32** | .02 |  |  |  |  | .51** | .03 |  |  |  |
| Education | -.08** | .02 |  |  |  |  | -.06** | .02 |  |  |  |
| Income | .06* | .03 |  |  |  |  | .03 | .02 |  |  |  |
| Rel. satisfaction | .00 | .02 |  |  |  |  | .00 | .02 |  |  |  |
| Number of children | .05* | .02 |  |  |  |  | .03 | .02 |  |  |  |
| Birth country | .27** | .06 |  |  |  |  | .14 | .07 |  |  |  |
| Child Age | .08** | .02 |  |  |  |  | .14 | .02 |  |  |  |
| Child Gender | -.01 | .03 |  |  |  |  | .03 | .04 |  |  |  |
| Birth order | -.01 | .02 |  |  |  |  | .03 | .03 |  |  |  |
| Negative emotionality | .01 | .02 |  |  |  |  | .00 | .02 |  |  |  |

*Note*. Rel. satisfaction. = Relationship satisfaction, Anx. & Depr. = Anxiety & Depression, Rec. supportive ESBs= Recollected supportive ESB, Rec. non-s. ESBs= Recollected non-supportive ESB, **p* < .01, ***p* < .001.

^a^ = including only a random intercept accounting for family-level clustering.
